# Weakly supervised deep learning to predict recurrence in low-grade endometrial cancer from multiplexed immunofluorescence images

**DOI:** 10.1038/s41746-023-00795-x

**Published:** 2023-03-23

**Authors:** Daniel Jiménez-Sánchez, Álvaro López-Janeiro, María Villalba-Esparza, Mikel Ariz, Ece Kadioglu, Ivan Masetto, Virginie Goubert, Maria D. Lozano, Ignacio Melero, David Hardisson, Carlos Ortiz-de-Solórzano, Carlos E. de Andrea

**Affiliations:** 1 Program of Solid Tumors and Biomarkers, Center for Applied Medical Research (CIMA), University of Navarra, Pamplona, Spain; 2 Department of Pathology, Clínica Universidad de Navarra, Pamplona, Spain; 3 Department of Pathology, Hospital Universitario La Paz, IdiPAZ, Madrid, Spain; 4 Navarra Institute for Health Research (IdISNA), Pamplona, Spain; 5 Lunaphore Technologies SA, Tolochenaz, Switzerland; 6 Akoya Biosciences, Marlborough, MA USA; 7 Center for Biomedical Research in the Cancer Network (CIBERONC), Madrid, Spain; 8 Department of Immunology and Immunotherapy, Clínica Universidad de Navarra, Pamplona, Spain; 9 Program of Immunology and Immunotherapy, Center for Applied Medical Research (CIMA), University of Navarra, Pamplona, Spain; 10 Molecular Pathology and Therapeutic Targets Group, La Paz University Hospital, IdiPAZ, Madrid, Spain; 11 Faculty of Medicine, Universidad Autónoma de Madrid, Madrid, Spain

**Keywords:** Machine learning, Image processing, Computer science, Predictive markers, Imaging the immune system

## Abstract

Predicting recurrence in low-grade, early-stage endometrial cancer (EC) is both challenging and clinically relevant. We present a weakly-supervised deep learning framework, NaroNet, that can learn, without manual expert annotation, the complex tumor-immune interrelations at three levels: local phenotypes, cellular neighborhoods, and tissue areas. It uses multiplexed immunofluorescence for the simultaneous visualization and quantification of CD68 + macrophages, CD8 + T cells, FOXP3 + regulatory T cells, PD-L1/PD-1 protein expression, and tumor cells. We used 489 tumor cores from 250 patients to train a multilevel deep-learning model to predict tumor recurrence. Using a tenfold cross-validation strategy, our model achieved an area under the curve of 0.90 with a 95% confidence interval of 0.83–0.95. Our model predictions resulted in concordance for 96,8% of cases (κ = 0.88). This method could accurately assess the risk of recurrence in EC, outperforming current prognostic factors, including molecular subtyping.

## Introduction

Histology-based morphological and architectural features of the tumor and its microenvironment are traditionally used to classify cancer, to prognose clinical outcomes, and to predict the response of patients to conventional or immune-based therapies^1–3^. This examination, primarily performed by a pathologist, uses histology with the aid of immunohistochemistry (IHC). However, despite the improvements in IHC testing and molecular profiling, the accuracy of predicting patient prognosis remains a current-day diagnostic challenge. In this context, multiplexed IHC/immunofluorescence (IF) methods provide new biological insights from conventionally used single-cell markers^4–8^. Multiplexed IF assays allow for the simultaneous visualization and quantification of several markers with single-cell resolution in a single tissue section. These assays have the immediate potential for translational research and clinical practice^4,9^. This is especially true when combined with new computational tools, such as deep learning, capable of generating computational tumor-immune maps, with the promise of improved and unbiased diagnostics^10–14^.

Advances in deep learning have increasingly demonstrated accurate and reliable performance in predicting patient outcomes from routine tumor tissue slides^12,15,16^. Among other methods, weakly-supervised deep learning (WSDL) stands out since it does not require manual expert annotations. It can be quickly applied in new unseen scenarios, showing an improved predictive performance when compared to traditional methods. WSDL offers a high-throughput, interpretable framework that can automatically, with no human intervention, extract morphological and architectural features typically not recognized by human experts^10,13^. NaroNet was the first WSDL framework to analyze multiplexed images^16^. NaroNet was developed based on the idea that the apparently chaotic distribution of cells in tissue sections can be transformed into highly structured microenvironmental tissue levels, known as (i) local phenotypes, (ii) cellular neighborhoods, and (iii) tissue areas. NaroNet has been previously used to develop a prediction model for the DNA polymerase epsilon (POLE) mutation in high-grade endometrial cancer^16^. The model had an overall accuracy of 83.3% and was trained and validated on multiplexed IF images using the spatial infiltration pattern of immune cells, the expression of the T-cell activation marker CD137 (4-1BB), and the programmed cell death-1 receptor (PD-1)^16^.

Endometrial cancer is the most common gynecologic malignancy in developed countries, where the majority of patients are diagnosed with low-grade, early-stage disease (FIGO stage I–II, G1-G2) with good clinical outcomes^17^. However, 5–10% of these patients will eventually experience tumor recurrence^17,18^. Due to the heterogeneity and complexity of these tumors, predicting the recurrence of endometrial cancer has been difficult^19^. Although several studies have shown the prognostic value of immune cell subtypes in high-grade endometrial cancer, particularly T cells and macrophages, there is still no evidence that a single marker alone could be used to improve risk stratification in patients with low-grade, early-stage disease^20,21^. Using a comprehensive multiplexed IF panel, we have recently shown that the tumor-immune microenvironment milieu is the most important factor for predicting tumor recurrence in low-grade, early-stage endometrial cancer, outperforming currently used prognostic variables, including molecular subtypes^22^.

Here, we have expanded our analysis from 235 patients to 250 patients with long-term follow-up and 32 tumor recurrences^22^. Our previous model was developed based on algorithms trained by experienced pathologists. Now, we developed a weakly-supervised, multilevel deep learning model that, without requiring manual expert annotation for regions of interest, could effectively predict recurrence from multiplexed IF images of primary resection samples of low-grade, early-stage endometrial cancer. The novelty here comes from the artificial intelligence nature of the approach. The WSDL model was developed using (i) our previously validated panel of seven markers (PD-L1, PD-1, CD8, CD68, FOXP3, CK, and DAPI)^22^, (ii) NaroNet, designed to extract the complex tumor-immune interrelations at multilevel features (local phenotypes, cellular neighborhoods, and tissue areas) using unsorted multiplexed IF images (in the current approach, no images were excluded from the analyses, while previously only images with a minimum tumor content of 20% were analyzed), and (iii) a tenfold cross-validation approach (in our previous approach, all samples were used to train the model). Lastly, we evaluated the robustness of the model and the power of NaroNet to overcome technical variabilities.

## Results

### Clinical characteristics and experimental overview

**Fig. 1:**
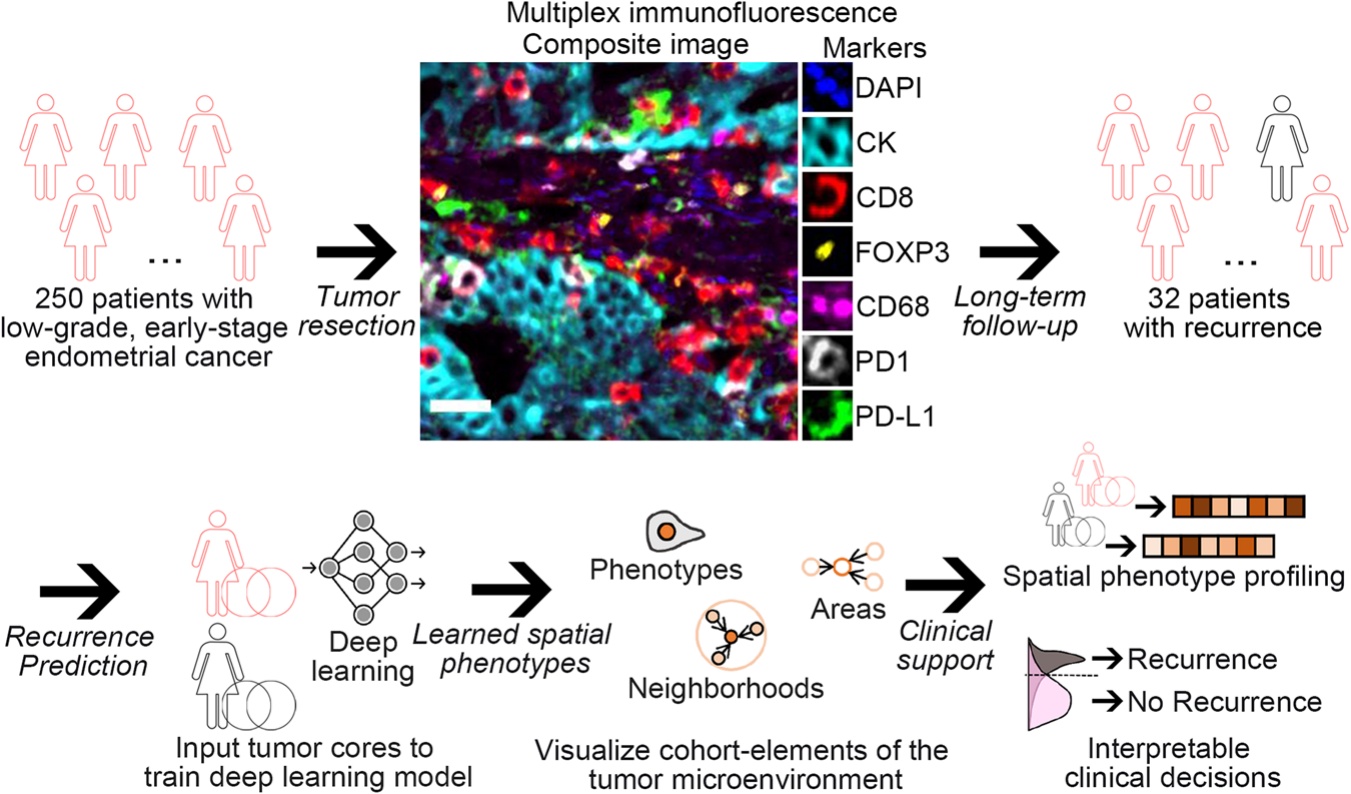
Artificial intelligence (AI)-based spatial phenotyping workflow to predict tumor recurrence in low-grade, early-stage endometrial cancer (EC). 250 patients with early-stage, low-grade endometrial cancer who underwent surgical resection were included in this study. Thirty-two tumors recurred over the follow-up period (mean 95.8 months). Tumor cores from primary tumor resection were stained using multiplexed immunofluorescence for the simultaneous visualization and quantification of CD68 of macrophages, CD8 + T cells, FOXP3 + regulatory T cells, PD-L1 and PD-1 protein expression, and tumor cells. NaroNet, a weakly-supervised deep learning framework, was applied to learn, without manual expert annotation, the complex tumor-immune interrelations at three levels: local phenotypes, cellular neighborhoods, and tissue areas. Our proposed method could accurately predict recurrence in EC, outperforming current prognostic factors, including molecular subtypes. Scale bar, 25 μm.

Our study included 250 eligible patients with low-grade, early-stage endometrial cancer (Fig. 1). The mean age of the patients was 64.5 years (Supplementary Table 1). Most patients completed at least 36 months of follow-up. Thirty-two tumors recurred over the follow-up period of 30.9 (18.3–50.5) months after surgery (Supplementary Fig. 1). Most patients were FIGO stage IA and G1 without lymphovascular invasion. Sixty-two percent of the patients received no adjuvant radiotherapy, while 27% of them received either external beam radiotherapy, vaginal brachytherapy, or both. Information about adjuvant radiotherapy was unavailable for 11.6% of the patients. Forty-five (18%) and eight (3.2%) patients showed mismatch repair protein deficiency and POLE mutations, respectively. Most tumors showed wild-type p53 expression by immunohistochemistry (96.8%, Supplementary Table 1).

A total of 489 multiplexed IF images were analyzed. Due to tissue loss during staining, 11 patients had one core available. Tumor cores from the same patient were merged into a single-image. For imaging preprocessing, background signal subtraction was applied using ImageJ software. No images were excluded from the analysis. Self-supervised learning for patch feature extraction was combined with weakly-supervised learning to enable a single, unified predictive model to be efficiently trained on hundreds of images. Using trainable assignment matrices, elements of the tumor microenvironment with high biologic relevance were identified and quantified. They were categorized as (i) local phenotypes, that link similar patches (20 × 20 µm) based on features derived from cell morphometry and marker expression, (ii) cellular neighborhoods, patches that aggregate features from adjacent patches via graph neural networks (GNNs) to form neighborhoods acquiring the contextual information in 100 × 100 µm, and (iii) tissue areas, where the graph of neighborhoods was used to aggregate neighborhood information in a way so that groups of neighborhoods form tissue areas. They are connected through edges representing the spatial affinities of the neighborhoods acquiring the contextual information in ~1800 × 1800 µm.

Within the NaroNet AI model, quantifications were passed through a max-sum pooling operation to obtain abundance values for each local phenotype, cellular neighborhood, and tissue area. Therefore, patients are represented by an enrichment vector containing abundance values of the microenvironmental elements. Enrichment vectors were then used to obtain the final patient prediction value. During training, a cross-entropy function loss updates model parameters to identify optimal tumor features for patient classification^23^.

NaroNet interpretability is based on heatmaps and graphs that summarize relevant features and patterns from the entire dataset^16^. This is achieved due to the previous assignment of patches to phenotypes, neighborhoods, and areas. Moreover, as patients are represented by abundance vectors, specific elements from the tumor microenvironment were associated with tumor recurrence.

### Evaluation of model performance

The initial self-supervised learning module transformed 20 × 20 × 7 image patches into representation vectors (each patch fits one or two cells and seven marker expressions). These vectors or embeddings of 256 values contain features from cell morphology, marker expression, and marker colocalization. When trained, self-supervised learning achieved an accuracy of 71.11%, contrasting a total of 8.520.336 image patches from the entire dataset, similar to what has already been reported in ref. ^24^. From these patch embeddings, a graph was created by connecting each image patch to its four adjacent neighbors and to itself. This graph represents the elements of the tissue microenvironment. Patient-level graphs were then generated by joining all the patch graphs available from one patient, which in general, consisted of one or two tumor cores.

**Fig. 2:**
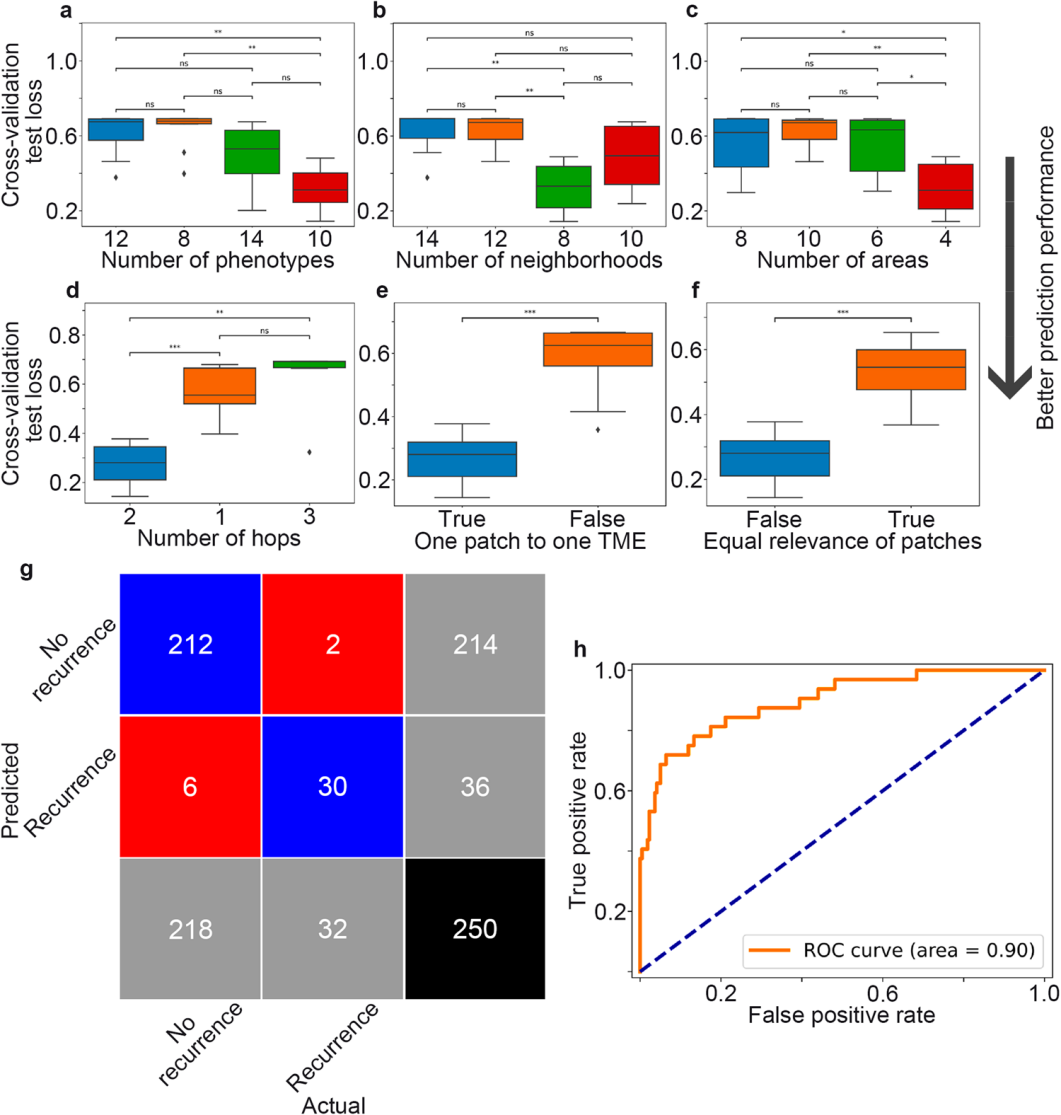
Evaluation of model performance. **a**–**c** Optimal number of local phenotypes, cellular neighborhoods, and tissue areas for patient classification. NaroNet had a better performance (lower cross-validation test loss) when set up to identify ten local phenotypes, eight cellular neighborhoods, and four tissue areas. **d** Optimal number of hops (levels of depth) used by the graph neural network for assigning patches to cellular neighborhoods. Two hops were selected to identify cellular neighborhoods (each patch was assigned to a cellular neighborhood based on its own embedding and those of its 12 closest neighbors) **e** NaroNet performed better when patches were constrained to be assigned to one tumor microenvironmental element (TME) only. **f** Every image patch had the same relevance in the classification process. **g**, **h** Confusion matrix and area under the curve of the receiver operating characteristic curve (AUC ROC) for predicting tumor recurrence in EC using a tenfold cross-validation strategy. Data were analyzed using a two-sided Mann–Whitney–Wilcoxon test with Bonferroni correction. *, **, ***: *P* < 0.05 values considered statistically significant. ns statistically non-significant.

By using self-supervised learning, the large input images were embedded in compact, low-dimensional, enriched patch graphs. Thus, NaroNet did not require long training times. As a result, our model hyperparameters were configured to provide the highest predictive performance using an architecture search algorithm called hyperparameter tuning (Supplementary Table 2). NaroNet had a better performance (lower cross-validation test loss) when set up to identify ten local phenotypes, eight cellular neighborhoods, and four tissue areas (Fig. 2a–c). Two levels of depth (two hops) were selected to identify cellular neighborhoods (each patch was assigned to a cellular neighborhood based on its own embedding and those of its 12 closest neighbors) (Fig. 2d). For the max-sum pooling operation, NaroNet performed better when patches were constrained to be assigned to one microenvironmental element only (a patch cannot belong to two local phenotypes simultaneously) (Fig. 2e). NaroNet also selected *softmax* over the *sigmoid* activation function so that every image patch had the same relevance in the classification process (patch contributions add up to one when obtaining patient representations) (Fig. 2f).

Finally, using the optimal NaroNet architecture, a tenfold cross-validation strategy was used to measure the classification performance for predicting tumor recurrence. In each fold, 225 patients (90%) were used to train the model, whereas the remaining 25 patients (10%) were used to test the model. Prediction performance was measured over the tenfolds with the ROC curve and confusion matrix shown in Fig. 2g, h. Even though moderate concordance rates between cores from the same patient were found (*r* = 0.6 for local phenotypes, *r* = 0.5 for cellular neighborhoods, *r* = 0.61 for tissue areas), our model predictions resulted in concordance for 96,8% of cases (κ = 0.88 when adjusted for agreement by chance). The overall accuracy was 90.40 with a 95% confidence interval (CI) of [86.72, 94.08] and an AUC of 0.90 with a 95% CI of [0.83, 0.95] (prediction values shown in Supplementary Table 3).

### Interpretability of local phenotypes

**Fig. 3:**
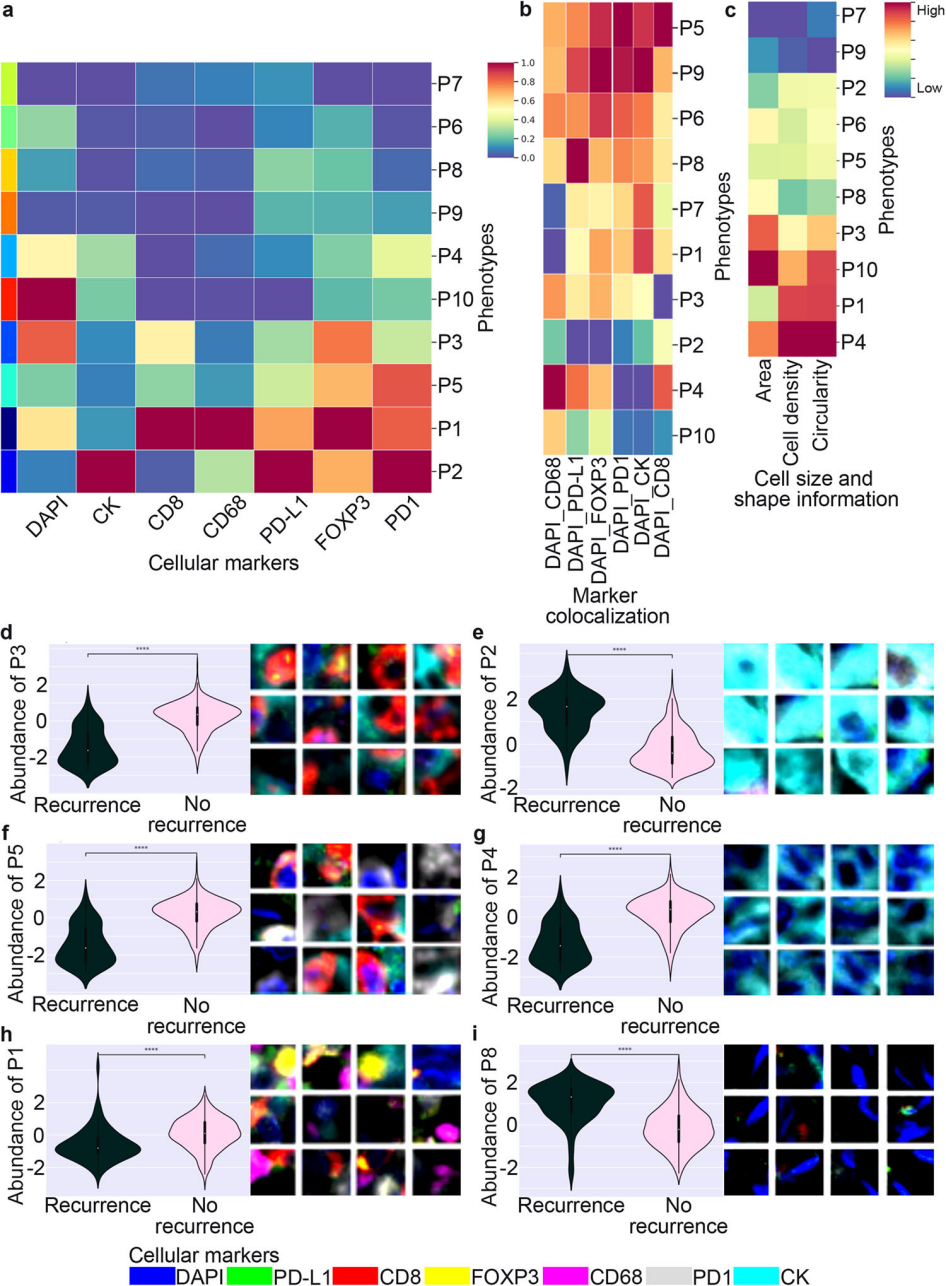
Interpretability of local phenotypes. **a** Heatmap showing the mean intensity for each marker in each one of the ten conserved, distinct local phenotypes (P1–10). For visualization, markers were normalized by columns; the maximum value for each marker is one. **b** Heatmap showing the level of colocalization between DAPI and other markers (calculated by Spearman correlation). For visualization, markers were normalized by columns; the maximum value for each marker is one. **c** Heatmap showing the cell morphology features (calculated based on cell segmentation for feature extraction: cell area, cell density, and cell circularity. For visualization, cell size and shape information vary from low to high. **d**, **f**, **h** The 12 most representative patches from each local phenotypes (P1, P3, and P5) associated with tumors with no recurrence were selected and displayed, allowing for a comprehensive interpretation of the entire dataset without the need to visualize full images individually. The relative abundance of each local phenotype is shown. **e**, **g**, **i** The 12 most representative patches from each local phenotypes (P2, P4, and P8) associated with tumors with recurrence were selected and displayed. The relative abundance of each local phenotype is shown. Data were analyzed using a two-sided Mann–Whitney–Wilcoxon test with Bonferroni correction. ****, *P* values: P1 = 2.23e-9, P2 = 5.95e-28, P3 = 3.94e-26, P4 = 2.94e-24, P5 = 6.67e-26, and P8 = 4.00e-20.

Ten conserved, distinct local phenotypes were found and measured (Supplementary Table 4). For each local phenotype, 100 patches (20 × 20 µm) with the highest confidence (most representative patches) were selected. The mean intensity for each marker is shown in a heatmap where columns were normalized. Thus, the maximum value for each marker is one (Fig. 3a). Two additional heatmaps were generated showing the level of colocalization between markers (calculated by Spearman correlation, Fig. 3b), and the cell morphology features (calculated based on Stardist^25^ cell segmentation masks and Morpholibj for feature extraction: cell area, cell density, and cell circularity, Fig. 3c)^26^.

The 12 most representative patches from each of the ten local phenotypes were selected and displayed, allowing for a comprehensive interpretation of the entire dataset without the need to visualize full images individually (Fig. 3d–h). Three local phenotypes, P1, P3, and P5, have captured the highest immune cell infiltrates and were more significantly associated with tumors with no recurrence (Fig. 3d, f, h). Local phenotype P1 was enriched with FOXP3 and CD68. Local phenotype P3 was enriched with CD8 and CK. Local phenotype P5 was enriched with CD8 and PD-1. In contrast, local phenotypes P2 and P4 captured different sizes of tumor cells alone (CK) with variable PD-L1 expression and no immune cell infiltration. Local phenotype P2 was more significantly associated with tumors with recurrence and local phenotype P4 was more significantly associated with tumors with no recurrence (Fig. 3e, g). Local phenotype P8 was enriched with cells with elongated nuclei expressing none of the markers (possibly stromal cells). Local phenotype P8 was also more significantly associated with tumors with recurrence (Fig. 3i).

### Interpretability of cellular neighborhoods

**Fig. 4:**
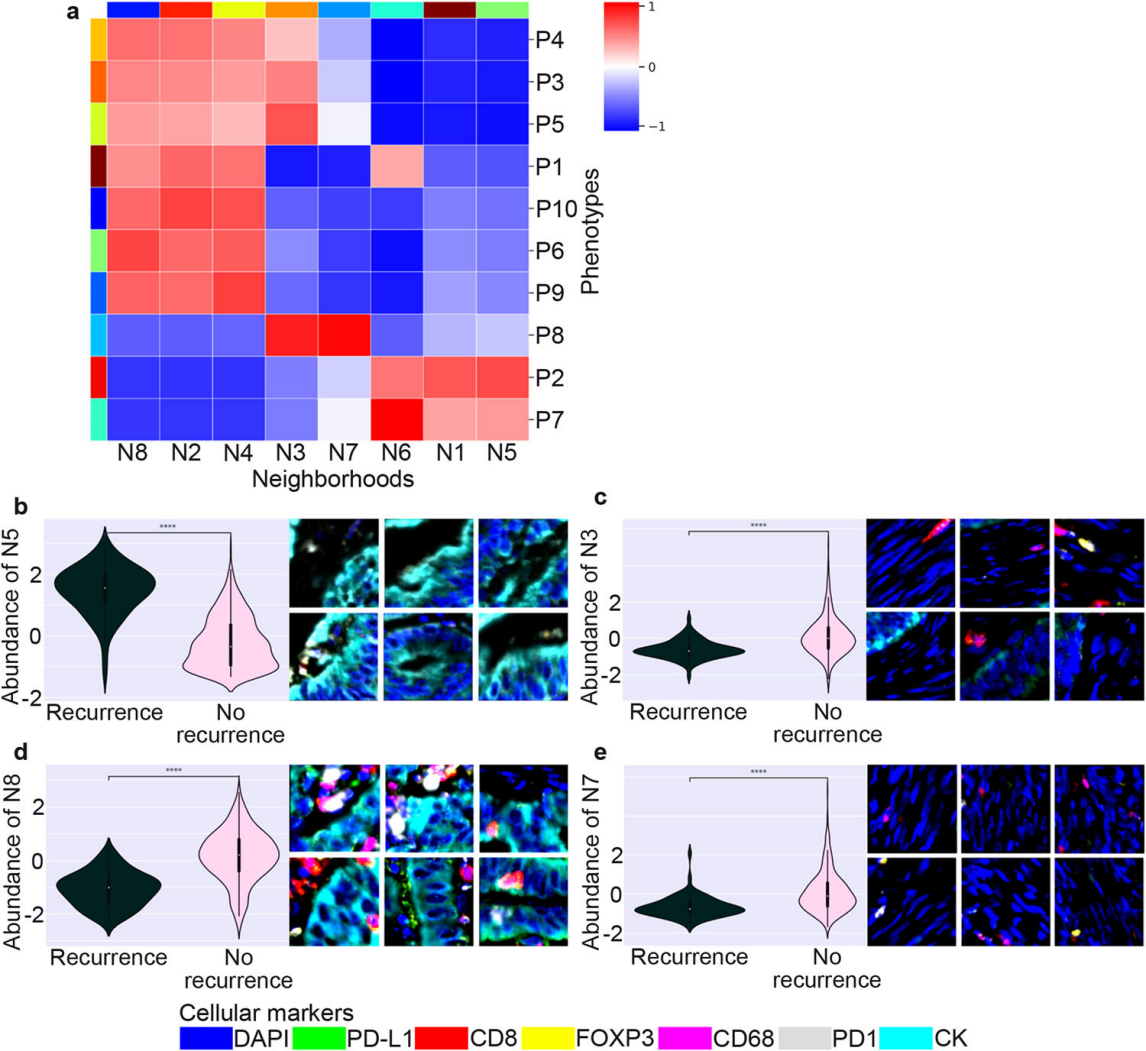
Interpretability of cellular neighborhoods. **a** Heatmap showing the eight conserved, distinct cellular neighborhoods (N1–8) composed of the proximity of the 12 most adjacent local phenotypes (P1–8). For visualization, relative abundances of local phenotypes were normalized by columns; the maximum value for each local phenotype in a neighborhood is one. **b** The six most representative images from cellular neighborhood N5. N5 was more associated with tumors with recurrence and represented regions enriched with tumor cells with variable PD-L1 expression and no immune cell infiltration. Relative abundance of cellular neighborhood N5 is shown. **c**–**e** The six most representative images from cellular neighborhoods (N3, N7, and N8) are more associated with tumors with no recurrence. N3 shows discrete immune cell infiltrates characterized by CD8, PD-1, FOXP3, CD68, and PD-L1. N7 is enriched with cells with elongated nuclei expressing none of the markers (possibly stromal cells) with discrete immune cell infiltrates. N8 shows higher immune cell infiltration with CD8, FOXP3, and CD68. Relative abundances of cellular neighborhoods N3, N7, and N8 are shown. Data were analyzed using a two-sided Mann–Whitney–Wilcoxon test with Bonferroni correction. ****, *P* values: N3 = 2.87e-11, N5 = 1.57e-29, N7 = 3.51e-12, and N8 = 2.58e-22.

Eight conserved, distinct cellular neighborhoods were found and measured (Supplementary Table 4). For each neighborhood, 100 patches with the highest confidence (the most representative patches) were selected and computed to which local phenotypes they were assigned. A heatmap was then generated. As cellular neighborhoods were calculated with two hops of distance, they were composed of the proximity of the 12 most adjacent local phenotypes (Fig. 4a).

Cellular neighborhood N5 contained mainly local phenotypes P2 and P7 and represented regions enriched with tumor cells with variable PD-L1 expression and no immune cell infiltration (Fig. 4b). Cellular neighborhood N5 was more significantly associated with tumors with recurrence. Cellular neighborhood N3 was composed of local phenotypes P3, P4, P5, and P8 which show few immune cell infiltrates characterized by CD8, PD-1, FOXP3, CD68, and PD-L1. Cellular neighborhood N3 was more significantly associated with tumors with no recurrence (Fig. 4c). Cellular neighborhood N8 was formed by local phenotypes P1, P3, and P5. It showed higher immune cell infiltration with CD8, FOXP3, and CD68 and was more significantly associated with tumors with no recurrence (Fig. 4d). Finally, cellular neighborhood N7 was mainly composed of local phenotype P8, which was enriched with cells with elongated nuclei expressing none of the markers (possibly stromal cells) with few immune cell infiltrates. Cellular neighborhood N7 was more significantly associated with tumors with no recurrence (Fig. 4e).

### Interpretability of tissue areas

**Fig. 5:**
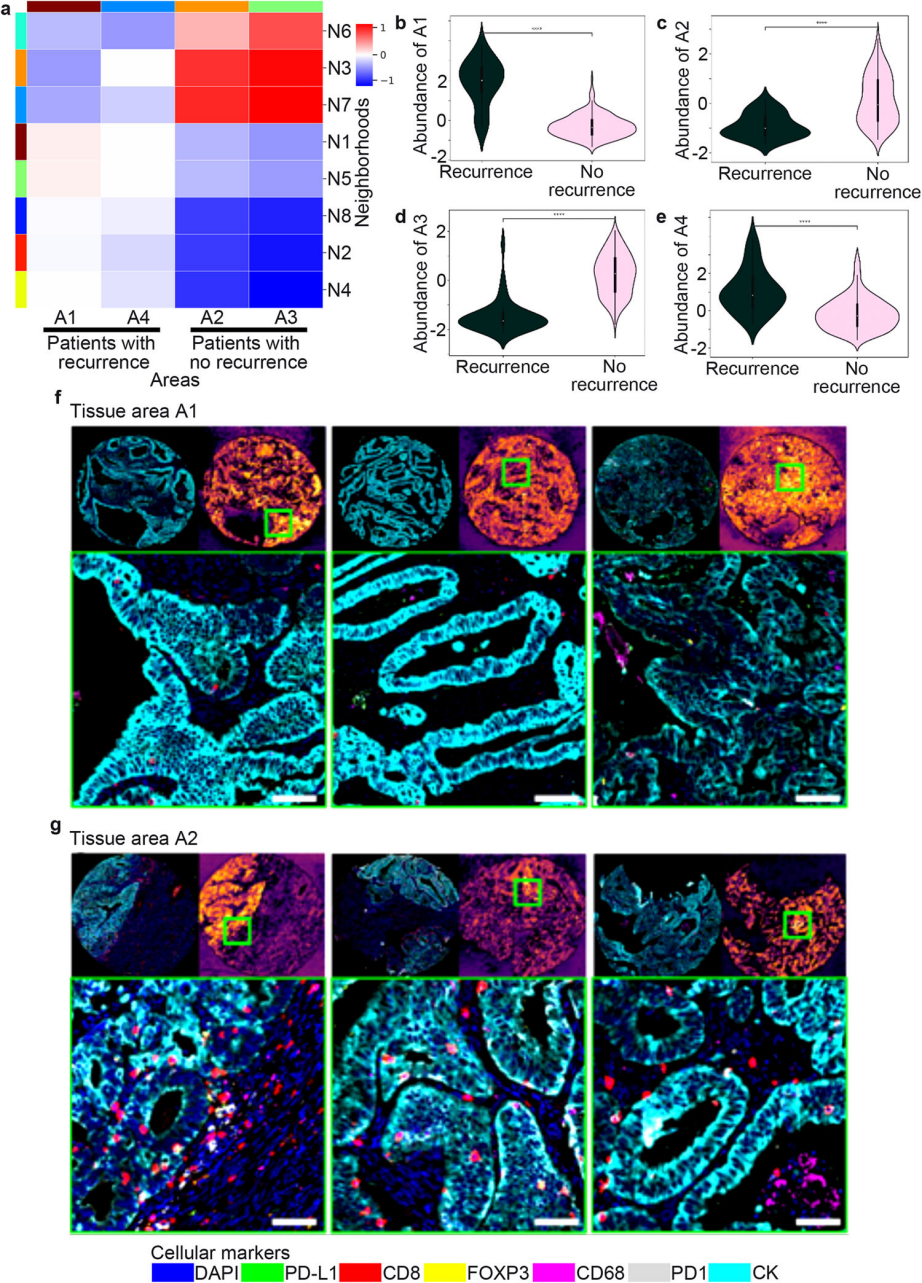
Interpretability of tissue areas. **a** Heatmap showing four conserved, distinct tissue areas (A1–4) composed of the spatially interacting cellular neighborhoods (N1–8). For visualization, relative abundances of cellular neighborhoods were normalized by columns; the maximum value for each cellular neighborhood in a tissue area is one. **b**, **e** Relative abundances of tissue areas A1 and A4 associated with tumors with recurrence are shown. **c**, **d** Relative abundances of tissue areas A2 and A3 associated with tumors with no recurrence are shown. **f** Top-3 tumor core images assigned to tissue area A1, which is associated with tumors with recurrence. For each one of the representative panel of images: the top left image shows the whole tumor core, the top right image shows the image patch confidence when assigning to tissue area, the bottom image shows a tumor region with the highest relevant information from tissue area A1 (more associated with tumors with recurrence). Tissue area A1 shows very little immune cell infiltration, representing “noninflamed” or “cold tumors”. **g** Top-3 tumor core images assigned to tissue area A2, which is associated with tumors with no recurrence. For each one of the representative panel of images: the top left image shows the whole tumor core, the top right image shows the image patch confidence when assigning to the tissue area, the bottom image shows a tumor region with the highest relevant information from tissue area A2 (more associated with tumors with no recurrence). Tissue area A2 is one of the most immune infiltrated, representing “inflamed” or “hot tumors”. Data were analyzed using a two-sided Mann–Whitney–Wilcoxon test with Bonferroni correction. ****, *P* values: A1 = 2.22e-29, A2 = 5.93-16, A3 = 6.25e-31, and A4 = 2.04e-12. Scale bars, 100 μm.

**Fig. 6:**
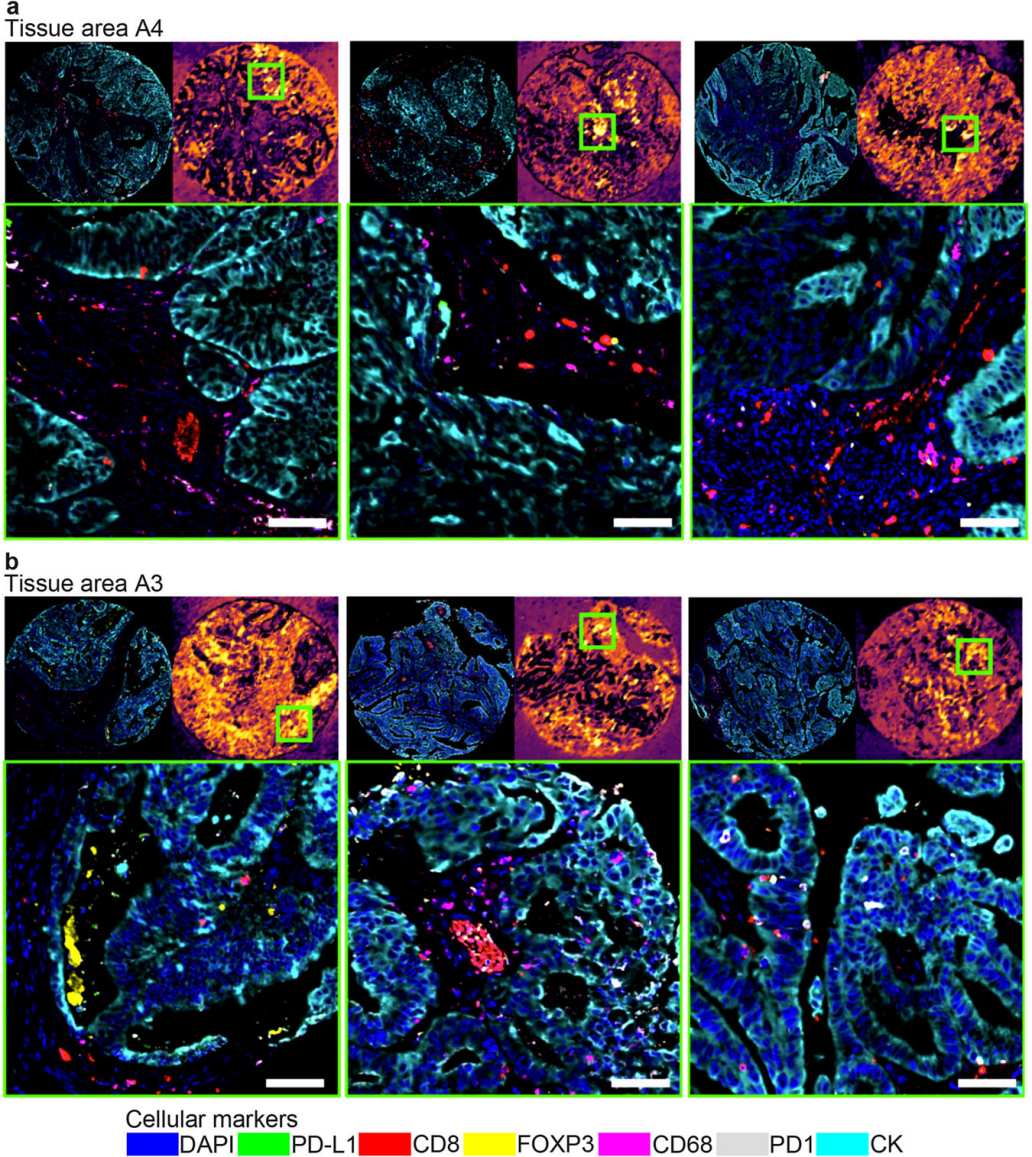
Visualization of tissue areas. **a** Top-3 tumor core images assigned to tissue area A4, which is associated with tumors with recurrence. For each one of the representative panel of images: the top left image shows the whole tumor core, the top right image shows the image patch confidence when assigning to tissue area, the bottom image shows a tumor region with the highest relevant information from tissue area A4 (more associated with tumors with recurrence). Tissue area A4 shows immune cell infiltration located in the stromal compartment, representing “noninflamed” or “cold tumors”. **b** Top-3 tumor core images assigned to tissue area A3, which is associated with tumors with no recurrence. For each one of the representative panel of images: the top left image shows the whole tumor core, the top right image shows the image patch confidence when assigning to tissue area, the bottom image shows a tumor region with the highest relevant information from the tissue area A3 (more associated with tumors with no recurrence). Tissue area A3 shows high immune cell infiltration located in the intra-tumor compartment, representing “inflamed” or “hot tumors”. Scale bars, 100 μm.

A total of four tissue areas were found and measured (Supplementary Table 4). Each tissue area was computed from the graph of cellular neighborhoods. Each neighborhood was assigned to a tissue area (Fig. 5a). Tissue areas A1 and A4 showed very little immune cell infiltration located in the stromal compartment, representing “noninflamed” or “cold tumors” (Figs. 5a, b, e, f, 6a). Tissue areas A1 and A4 were more significantly associated with tumors with recurrence. Tissue areas A2 and A3 were the most immune infiltrated with intra-tumor CD8 + T cells located. Tissue areas A2 and A3 were more associated with tumors with no recurrence, representing “inflamed” or “hot tumors” (Figs. 5a, c, d, g, 6b).

### Evaluation of the clinical characteristics and molecular subtypes

Tumor grade, patient age, and p53 status showed no association with any of the local phenotypes, cellular neighborhoods, or tissue areas. In contrast, most of the morphological patterns found by NaroNet were associated with FIGO stage and lymphovascular invasion (Supplementary Fig. 1). Tissue area A2 was significantly underrepresented in mismatch repair protein (MMRP)-deficient tumors compared to their proficient counterparts. Tissue area A2 showed high immune cell infiltration and was more associated with tumors with no recurrence. Furthermore, local phenotypes P1 and P3 were significantly enriched in POLE-mutated tumors, while N5 and P7 were underrepresented in these tumors (Supplementary Fig. 1). Local phenotype P1 showed high immune cell infiltrates with CD8, FOXP3, and CD68. Local phenotype P3 represented the local interaction between a CD8 T lymphocyte and tumor cells (CK). Local phenotypes P1 and P3 were more associated with tumors with no recurrence. Local phenotype P7 and cellular neighborhood N5 showed regions enriched with tumor cells with no immune cell infiltration and were associated with tumors with recurrence. Finally, NaroNet’s prediction model was compared to those built using mismatch repair protein status and the presence of a POLE mutation. The NaroNet model (AUC = 0.90) outperformed the other two models (AUC = 0.79 and 0.78).

### NaroNet robustness

Multiplexed IF staining was performed in two different institutions using a standardized and validated protocol. Nine TMAs were stained at the University of Navarra, and five TMAs were stained at Lunaphore Technologies. Each institution stained their allotment of slides with distinct staining batches (antibodies and reagents). Therefore, potential batch-to-batch variation across institutions was expected.

**Fig. 7:**
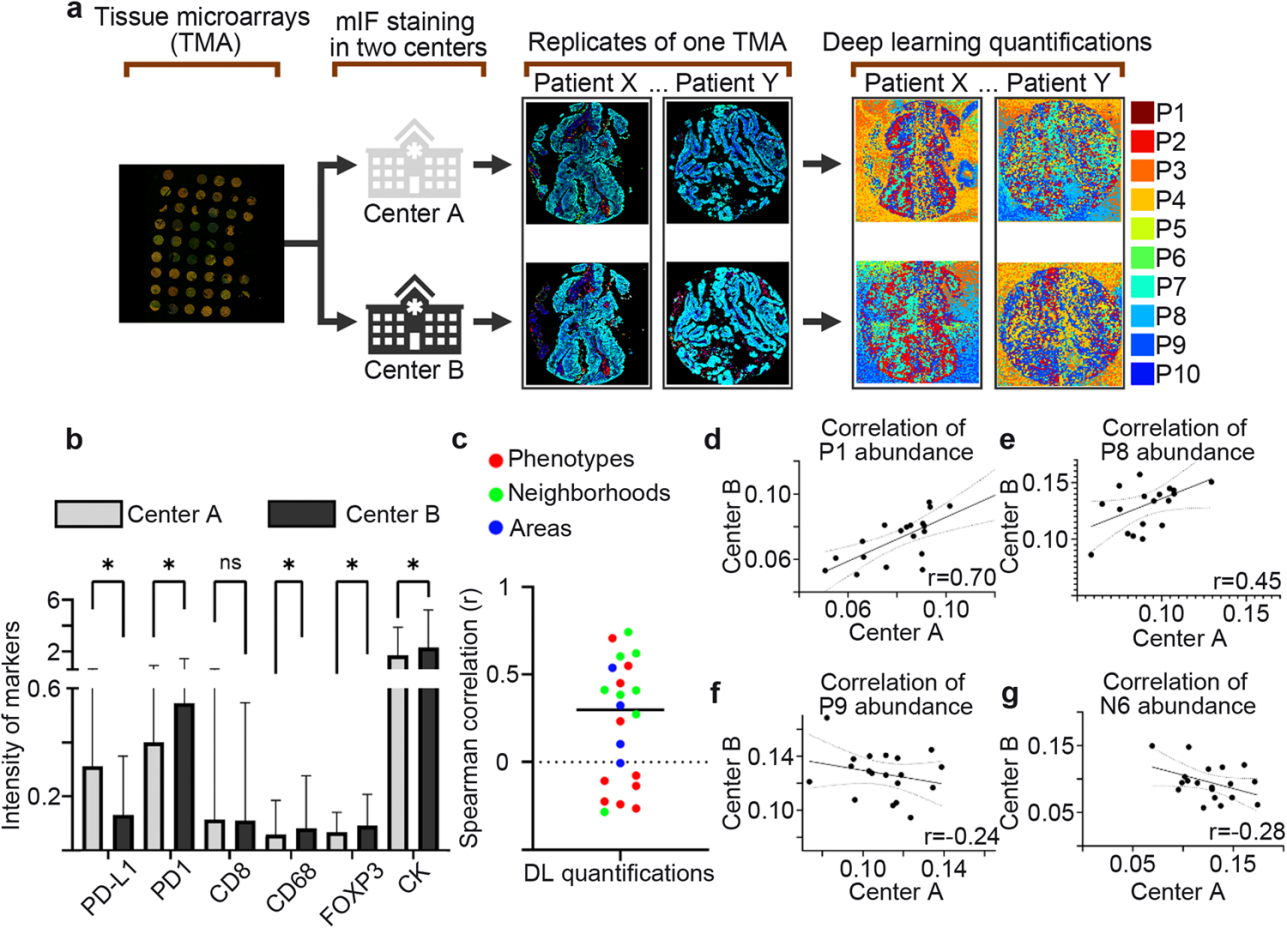
NaroNet robustness. **a** Multiplexed immunofluorescence staining on tissue microarrays (TMAs) was performed in two different centers using a standardized and validated protocol. Nine TMAs were stained at the University of Navarra, and five TMAs were stained at Lunaphore Technologies. To obtain a reference for variability between cases, one TMA with 36 tumor cores from 19 patients was stained in both institutions. In the replicate cores, deep learning quantifications were performed. **b** In the replicate cores, intensity measurements for each marker were then calculated. No threshold normalization was applied to adjust for potential batch-to-batch variation. As expected, the intensity variation is found across centers. **c** The overall quantification of the relevant local phenotypes, cellular neighborhoods, and tissue areas showed good correlations. **d**, **e** Relevant elements, such as local phenotypes P1 and P8, showed positive correlations across institutions. **f**, **g** Negative correlations were found only for the microenvironmental elements located in regions with no tissue (background staining), such as local phenotype P9 and cellular neighborhood N6. *r*, Spearman correlation coefficient with either positive value indicates positive association and negative value shows a negative association. *P < 0.05 values considered statistically significant. ns statistically non-significant.

To obtain a reference for variability between cases, one TMA with 36 tumor cores from 19 patients was stained in both institutions (Fig. 7a).

In the replicate cores, nuclear segmentation was performed using the DAPI channel. Each segmented nucleus was then expanded to provide an approximation of the full cell area. Intensity measurements for each marker were then calculated. No threshold normalization was applied to adjust for potential batch-to-batch variation. As expected, intensity variation was found across institutions (Fig. 7b). Similar variation using a similar multiplexed IF assay has been previously reported in a multi-institutional study^4,7^. The most significant variation was found in PD-L1 staining (*p* < 0.0001). CD8 staining showed similar intensity across institutions (*p* > 0.05).

Although intensity variations were found, the overall quantification of the relevant local phenotypes, cellular neighborhoods, and tissue areas showed good correlations (Fig. 7c). More importantly, tumors stained in both institutions were used to efficiently train and test our model predictions. Our model predictions resulted in concordance in 92 and 85% in both institutions separately, showing that, to some extent, NaroNet is capable of generalization to diverse data sources. Moreover, relevant elements, such as local phenotypes P1 and P8, showed positive correlations across institutions (*r* = 0.70 and 0.45, respectively, Fig. 7d, e). In contrast, negative correlations were found only for the microenvironmental elements located in regions with no tissue (background staining), such as local phenotype P9 and cellular neighborhood N6 (Fig. 7f, g). This shows that NaroNet captured differences in background staining and assigned it to nonrelevant microenvironmental elements.

### Analysis of prediction confidence

Using only two tumor cores from primary tumor resections to predict patient recurrence is very challenging. They may not represent the totality of the tumor. Here, our model predictions classified the vast majority of tumors correctly (confidence of 0.90). This suggests that the primary tumors were well represented in the TMAs. In a subset of these patients, we have previously shown a very good correlation between the immunofluorescence results of TMAs and whole-slide tumor sections^22,27^.

**Fig. 8:**
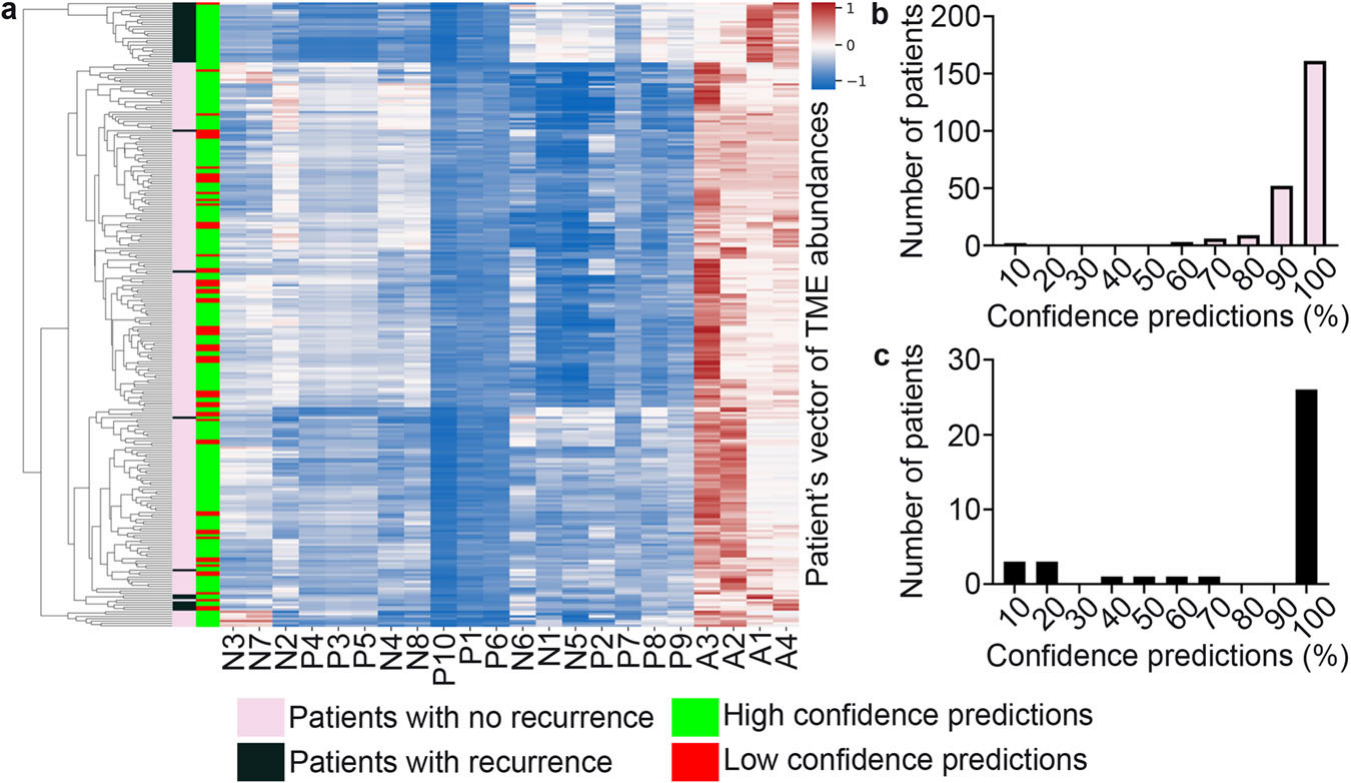
Analysis of prediction confidence. **a** Heatmap showing the “prototypical” spatial features of the tumor microenvironmental elements (TME) at three levels: local phenotypes (P1–10), cellular neighborhoods (N1–8), and tissue areas (A1–4) in tumors with recurrence and tumor with no recurrence. High and low-confidence predictions are shown. **b**, **c** The overall confidence predictions of NaroNet for all 250 patients is shown. The vast majority of patients show high confidence predictions (>0.90). Patients with low-confidence predictions (<0.50) were misclassified.

**Fig. 9:**
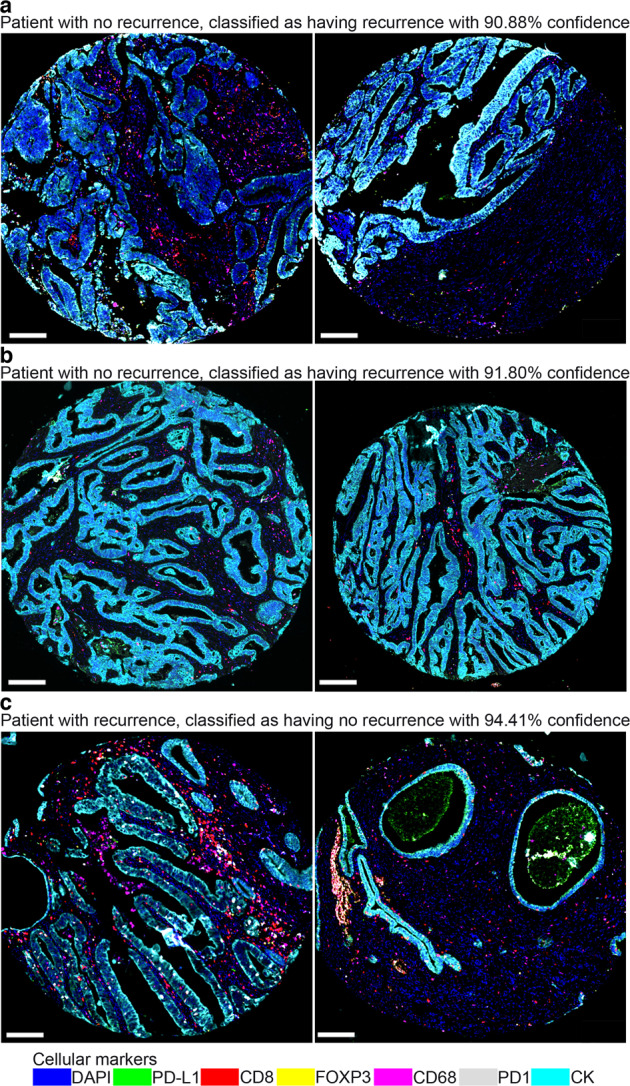
Evaluation of challenging patients. **a**, **b** Only two patients with no recurrence were incorrectly classified as having recurrence (confidence predictions >0.90). They developed late endometrial cancer relapses after >7 years of relapse-free survival. Both patients were quantified as tissue area A1, indicating “noninflamed” or “cold tumors”. Visual interpretation of the tumor cores showed heterogeneity within the tumor. **c** Patients with recurren**c**e that were incorrectly classified as having no recurrence (confidence prediction >0.90) showed tumor cores quantified as tissue area A3, indicating “inflamed” or “hot tumors”. Visual interpretation of the tumor cores showed inflammation and distinct glandular architectural patterns. Scale bars, 50 μm.

Looking at the complex tumor-immune interrelations for all patients (Fig. 8a–c), tumors with recurrence showed the “prototypical” spatial features of “noninflamed” or “cold tumors” represented by tissue areas A1 and A4. Next, we looked at the tumors misclassified by NaroNet with a high prediction confidence. Only two tumors with no recurrence were incorrectly classified as having recurrence (confidence >0.90, Fig. 9a, b). They developed late endometrial cancer relapses at 86 and 90 months after resection (Supplementary Fig. 2). Tumor cores from these two patients were quantified as tissue area A1, indicating “noninflamed” or “cold tumors”. Visual interpretation of the tumor cores showed heterogeneity within the tumor (Fig. 9a, b). Furthermore, tumors with recurrence that were incorrectly classified as having no recurrence (confidence >0.90, Fig. 9c) showed tumor cores quantified as tissue area A3, indicating “inflamed” or “hot tumors”. Visual interpretation of the tumor cores showed inflammation and distinct glandular architectural patterns (Fig. 9c). The incorrect classifications show that tumors are complex diseases and suggest that, for some cases, predictions based solely on a panel of seven cellular markers are not sufficient.

### Support for clinical decisions

**Fig. 10:**
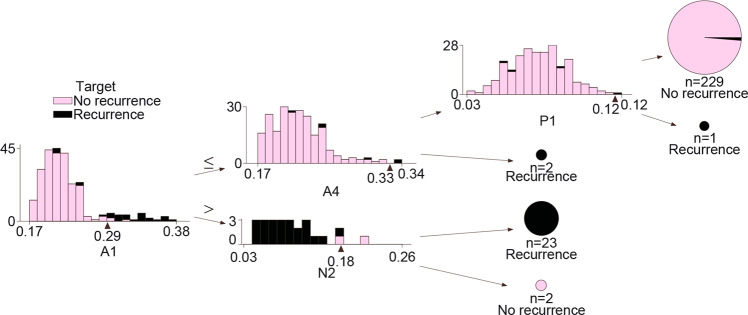
Support to clinical decisions. Decision tree to assess patient recurrence based on spatial phenotype quantifications. In each decision tree step (from left to right), an automatic threshold is set to divide patients with recurrence from those with no recurrence. Tissue area A1 (“noninflamed” or “cold tumors”) was the most relevant spatial feature to differentiate between tumors with recurrence from those with no recurrence.

Finally, we set up a patient screening process by implementing a decision tree in which spatial phenotype quantifications were used to make the final patient prediction. Here, a decision tree regressor was fed by the spatial features of all patients. Figure 10 shows the optimized scheme of the decision tree, which was based on three sequential steps. Tissue area A1 (“noninflamed” or “cold tumors”) was the most relevant spatial feature to differentiate between tumors with recurrence from those with no recurrence.

## Discussion

Predicting recurrence in low-grade, early-stage endometrial cancer is both difficult and important. Recurrence is seen in 5–10% of patients, and recurrence outside the pelvis is typically untreatable. Different studies have demonstrated the importance of molecular subtypes in the clinical outcomes of high-grade endometrial cancer. Most of these subtypes are associated with spatial immune infiltration patterns in the tumor. Despite the prognostic value of immune infiltration in endometrial cancer, relatively little is known about whether these immune features could accurately be associated with the risk of recurrence in patients with the low-grade disease. Recently, we have shown that the immune infiltration features in a primary tumor resection studied with multiplexed IF (PD-L1, PD-1, CD8, CD68, FOXP3, and CK) could reliably predict recurrence in patients with low-grade, early-stage endometrial cancer, outperforming the molecular subtypes^22^. Here, we have confirmed this.

It has been shown that the type of immune response may play a role in preventing tumor recurrence^28,29^. Recent studies have shown the prognostic value of immune cell infiltration in clinical outcome and treatment response in endometrial cancer^19–21^. Tumor-associated macrophages have been linked to a high relapse-free survival rate after surgery^20^. Higher CD8 + T-cell infiltration has been shown to be an independent predictor of improved overall survival^21^. Moreover, a high CD8+/FOXP3+ ratio has been shown to be an independent prognostic factor for better disease-free survival^21^. However, the identification of predictive biomarkers consistently associated with recurrence derived from a comprehensive multiplexed IF assay has not been thoroughly studied. Here, we follow the pioneering efforts of pivotal research groups to use multiparametric assays on single tissue sections to characterize the tumor microenvironment of solid tumors^2,30–32^.

Our weakly-supervised, multilevel deep learning model was used, without manual expert annotation, to train a model on a large and well-characterized collection of 489 TMA tumor cores from 250 patients with low-grade, early-stage endometrial carcinoma. This represents a unique set of tumors with long-term follow-up and clinical annotation. Quantitative multiplexed IF targeting the PD-L1 and PD-1 protein levels, CD68 + macrophages, CD8 + T cells, FOXP3 + regulatory T cells, and CK + tumor cells was used to assess the association between the complex tumor-immune interrelations and the risk of recurrence. To create a tumor recurrence prediction model, our algorithm trained graph neural networks with only patient recurrence data labels. Using a 10-fold cross-validation strategy (in each fold, 90% of patients were used to train the model and 10% of patients were used to test the model), an overall prediction value of 90.40% with a 95% CI of [86.72, 94.04] and an AUC of 0.90 with a 95% CI of [0.83, 0.95] were achieved. Our multilevel interpretable deep learning framework allowed the model to learn three levels of interactions within tissues: (i) local phenotypes (one single cell or interactions between two neighboring cells), (ii) cellular neighborhoods, and (iii) tissue areas. These features are of high biologic relevance and combine information to make the final predictions of tumor recurrence. Each level of interaction and feature in the deep learning framework is fully interpreted using graphs, heatmaps, and direct visualization in the tissue.

At the local phenotype level, NaroNet showed tumor-immune cell interactions that were positively and negatively associated with recurrence in patients with low-grade, early-stage endometrial cancer. Tumors with no recurrence were more inflamed. Their tumor microenvironment was characterized by immune cells closely interacting with tumor cells. This immune infiltration was composed of FOXP3 + regulatory T cells in close contact with either macrophage (local phenotype P1) or CD8 + T cells (local phenotype P3) and CD8 + T cells expressing PD-1 (local phenotype P5). Tumors with recurrence were less inflamed. Their tumor microenvironment was characterized by tumor cell enrichment with variable PD-L1 expression (local phenotype P2) or with no infiltration of immune cells (local phenotype P8).

It has been proposed that mutual information relationships provided by cell marker expression and cell localization within tissue reveal the spatial organization of the tumor microenvironment, known as cellular neighborhoods^31,33^. Here, the cellular neighborhoods are composed of neighboring local phenotypes. In our interpretable deep learning framework, for every local phenotype in tissue, its 12 nearest spatial neighbors are identified and labeled as cellular neighborhoods. We found eight conserved, distinct cellular neighborhoods. The information relationships that link each cellular neighborhood were further identified and interpreted as tissue areas. Each tissue area could recapitulate the tumor tissue. The relationship between cellular neighborhoods and tissue areas was then used in the multilevel process to predict disease recurrence in patients with low-grade, early-stage endometrial cancer. Cellular neighborhood N3 was associated with tumors with no recurrence and was enriched for FOXP3 + regulatory T cells, CD8 + T cells, high PD-1 protein levels, stromal cells, and tumor cells. Cellular neighborhood N3 is mainly composed of local phenotypes P1, P3, and P5. Next, cellular neighborhood interactions, known as tissue areas, provide a novel, interpretable view of patient samples. Integrating tumor and immune cell neighborhoods, NaroNet showed that tumors with recurrence were composed of tumor islands within a stromal cell-enriched stroma with few immune cells (tissue area A1), indicating “noninflamed” or “cold tumors”. Conversely, tissue areas A2 and A3 displaying FOXP3 + regulatory T cells and CD8 + T cells with high PD-1 protein levels interacting with tumor cells were strongly associated with no recurrence, indicating “inflamed” or “hot tumors”. Tissue area A1 contains the most relevant feature to differentiate between tumors with recurrence from those with no recurrence. Tissue area A1 can be interpreted as tumors with a “noninflamed” or “cold” microenvironment.

Recent studies have demonstrated that deep convolutional neural networks applied to H&E-stained histopathology images were able to predict the histological and molecular subtypes and common gene mutations in endometrial carcinoma^34^. Examining H&E slides is still the most widely used method by pathologists to assess endometrial cancer histological subtypes in the clinical setting. Therefore, WSDL models have shown great potential in assisting pathologists in making decisions and improving diagnostic accuracy^12,13,35^. While this article was under review, using routinely H&E-stained slides and molecular and clinicopathological from 2028 patients with intermediate-to-high-risk endometrial cancer, the group of Fremond S. et. al. reported a deep learning model for H&E-based prediction of cancer classification that could accurately identify morpho-molecular correlates outperforming the molecular classification at predicting 5-year recurrence-free survival in an independent cohort (*n* = 393)^36^. These results are in agreement with our main conclusions, though the use of multiplexed IF may strengthen the performance of the predictions.

Here, we used multiplexed IF, which allows for the simultaneous visualization of several cellular markers in a single tissue section, giving a more comprehensive understanding of the complex tumor-immune interrelations. In precision immuno-oncology, these interrelations have been used to identify prognostic and predictive biomarkers^37^. Standardization and validation of an end-to-end multiplexed IF workflow is critical^7^. We assessed the reproducibility of NaroNet to input data from two institutions using a similar and standard workflow. Although the intensity of the markers varied, the quantification of the relevant local phenotypes, cellular neighborhoods, and tissue areas could still capture critical microenvironmental elements allowing a very high predictive ability independent of the center of origin. It shows robustness. The relatively higher interinstitution variations were likely due to batch-to-batch variation and the local handling of assay reagents, such as how accurately they were prepared or diluted. These sources of variability have been previously reported in multiplexed IF, which can be further improved with additional standardization of reagent and instrument handling^4,7,8^.

The outstanding predictive performance obtained by NaroNet (90.40%) showed that two tumor cores per patient could be used to correctly predict the risk of recurrence. In our framework of analysis, they were merged into a single image. The modest concordance rates between tumor cores from the same patient highlight that the spatial heterogeneity of the tumor architecture and immune infiltrates are highly variable across tumor lesions. In this line, complex tumors displaying higher tumor-immune heterogeneity could be enriched in more aggressive tumor subclones that could potentially be linked to the propensity for recurrence. It must be acknowledged that is very difficult to quantitatively measure spatial heterogeneity with only two tumor cores.

Tumors with recurrence displayed a common architecture of tissue areas A1 and A4, and tumors with no recurrence showed a common architecture of tissue areas A2 and A3. Tumors with recurrence misclassified as having no recurrence displayed some features of tissue areas A2 and A3 that are more associated with tumors with no recurrence. This incorrect prediction is unlikely due to incorrect AI quantification but possibly due to other factors that were not considered in the analysis, reflecting the complexity of these tumors. Other factors that could have played a role in misclassifying tumors are the heterogeneous immune environment, the mutational profile, and other characteristics intrinsic to the patient (such as comorbidities, age, etc.). Importantly, the two tumors with no recurrence that were classified as recurrence developed very late endometrial cancer relapse at 86 and 90 months after primary tumor resection. These very late disease recurrences after >7 years of relapse-free survival show an indolent tumor behavior. Therefore, it is of the highest clinical interest to combine patient characteristics obtained from different sources. They might be relevant and relatively easy to assess. In this way, our AI quantifications (local phenotypes, cellular neighborhoods, and tissue areas) could be incorporated into multi-omics data analysis pipelines to increase patient prediction and better understand the complexity of the disease.

In summary, we developed a predictive model based on the spatial morphologic immune cell composition associated with the risk of recurrence in low-grade, early-stage endometrial cancer. Our WSDL interpretable approach automatically learned to locate regions in the TMA tumor core by local phenotypes, cellular neighbors, and tissue areas and to combine their information to make the final prediction. Combining AI-based models with multiplexed IF has the potential to unveil novel tumor-immune interrelations that human experts traditionally would not focus on. Future work may benefit from a more comprehensive study analyzing the composition and spatial distribution of immune cells using many more cellular markers in independent cohorts of low-grade, early-stage endometrial cancer.

## Methods

### Patient cohort and tissue microarrays

Formalin-fixed, paraffin-embedded (FFPE) samples from a retrospective collection of low-grade (FIGO G1 or G2) endometrioid carcinomas presented in tissue microarrays (TMAs) were analyzed. This included samples seen at the Pathology Department of the University Hospital La Paz (Madrid, Spain) from 250 patients with low-grade endometrioid carcinomas that met the following inclusion criteria: (i) surgical treatment and long-term follow-up undertaken at the University Hospital La Paz; (ii) all tumors were low-grade (G1 or G2) endometrioid carcinomas, stages I and II, according to the 2009 International Federation of Gynecology and Obstetrics (FIGO) classification; (iii) patients did not undergo neoadjuvant/adjuvant systemic treatment or immunotherapy; and (iv) because most tumors recur in the first 3 years after diagnosis, all patients had a minimum follow-up period of 3 years. All molecular subtypes were included in the analysis. This collection has been reported previously^22,27^. All tissues were used after approval from the University Hospital La Paz Human Research Committee, protocol number: HULP: PI-3108. TMAs were constructed using standard procedures as described previously in refs. ^38,39^. In brief, after a pathology review of hematoxylin and eosin (H&E)-stained slides, 1.2-mm cores were obtained from the original paraffin blocks using a needle and inserted into a recipient paraffin block. For a better representation of the tumors, two cores obtained from different areas were included in the TMAs. Clinicopathologic information from all patients, including disease recurrence, was collected from clinical records and pathology reports and is detailed in Supplementary Table 1. Patient follow-up was completed after 10 years.

### Multiplexed immunofluorescence staining

Multiplexed IF assay development and validation have been previously described by our group^5,38^. A seven-color multiplexed quantitative immunofluorescence protocol for FFPE tissue sections was used for simultaneous detection of CD8, FOXP3, CD68, PD-1, PD-L1, CK, and DAPI. Of note, since each section is put through seven sequential rounds of antibody staining, tyramide signal amplification-based (TSA) visualization is expected to detect a slightly higher number of positive cells. Therefore, each single antibody was optimized individually for its optimal conditions and position in the sequence of multiplex staining to reduce interference with previous antibody-TSA complexes or by disruption of epitopes. Single stains (singleplex) were then initially performed on endometrial carcinoma tissue sections (positive controls). Singleplex assays were used as the gold standard for cell antigen visualization. The markers were then integrated into a multiplexed immunofluorescence panel. Each single antibody was optimized individually for its optimal conditions and position in the multiplexed staining sequence. A singleplex versus multiplexed comparison for each antibody was performed to validate the staining patterns and distribution. Based on this comparison, we established the optimal signal through dilution of the primary antibodies and/or the fluorophores to obtain staining levels and cell counts comparable to conventional singleplex immunofluorescence staining as previously described^5^. Although staining variation was observed between singleplex and multiplex, overall, the markers had similar cell counts as previously described in ref. ^22^. Respective immunostainings without primary antibodies on endometrial carcinoma tissue sections were used as negative controls.

Multiplexed immunofluorescent staining was performed on the LabSat® Research platform (Lunaphore Technologies), a fully automated tissue-staining instrument for rapid immunostaining that utilizes a microfluidic technology for the rapid and uniform delivery of reagents to tissue samples^40^. In brief, TMA sections were dewaxed, rehydrated, and subjected to heat-induced antigen retrieval with a high pH (pH 9, catalog # S2367) target retrieval solution (Dako-Agilent). Each TMA section was subjected to six successive rounds of antibody staining. The antibody panel included cytokeratin (pan-CK) (1:150 clone AE1/AE3, catalog # NBP2-33200, Novus Biologicals), CD8 (1:150, clone 4B11, catalog # MCA1817, Bio-rad), CD68 (1:75, clone PG-M1, catalog # M0876, Dako-Agilent), FOXP3 (1:50, clone 236 A/E7, catalog # ab252201, Abcam), PD-1(1:300, ERP4877, catalog # ab137132, Abcam), and PD-L1 (1:300, clone E1L3N, catalog # 13684, Cell Signaling). Each round consisted of protein blocking with 20% normal goat serum (catalog # X0907, Dako-Agilent) in phosphate-buffered saline (PBS), incubation with the primary antibody, biotinylated anti-mouse/rabbit secondary antibodies and streptavidin-HRP (catalog # P0397, Dako-Agilent), followed by TSA visualization with fluorophores Opal 520, Opal 540, Opal 570, Opal 620, Opal 650, and Opal 690 (catalog # NEL861001KT, Akoya Biosciences) diluted in 1X Plus Amplification Diluent (catalog # FP1609, Akoya Biosciences). Thus, in the seventh round, nuclei were counterstained with spectral DAPI (catalog # FP1490, Akoya Biosciences), and sections were mounted with Faramount Aqueous Mounting Medium (catalog # S3025, Dako-Agilent). In our experimental setup, 9 TMAs were stained in one center, and another 5 TMAs were stained in another. For comparison, one TMA with 36 tumor cores was stained in both centers using the same platform and protocol but with distinct batches of antibodies and reagents.

### Tissue imaging

Multiplexed immunofluorescence TMA slides were scanned on a PhenoImager HT Automated Quantitative Pathology Imaging System (Akoya Biosciences). Briefly, a spectral library containing the spectral peaks emitted by each fluorophore from single stained slides was created using inForm software (version 2.4.8, Akoya Biosciences). This spectral library was used for spectral unmixing of the images, allowing color-based identification of the markers of interest. Autofluorescence was determined on an unstained endometrial carcinoma tissue. Each TMA core image was spectrally unmixed and exported as a component TIF image (2656 × 2656 × 7 pixels) using Akoya Biosciences’ Inform software. Component TIF images were then imported into the NaroNet deep learning framework. Due to tissue loss during multiplexed staining, each patient contributed one or two TMA tumor cores.

### Image preprocessing

Each image was preprocessed with background subtraction using the ‘rolling ball’ algorithm in ImageJ software version 1.52c (NIH, Bethesda, MD, USA; http://imagej.nih.gov/ij)^41^. Here, a local background value was determined using a window of 50 × 50 pixels. This value was then subtracted from the original image, removing large spatial variations in the background intensities. The window size was set as the size of the largest cell object that is not part of the background, so that the real marker signal was not eliminated.

### Patch feature extraction

In our WSDL framework, two tumor core images from the same patient were merged into a single image. Then, pixel information from each image was converted into a representation space, enabling a drastically faster training time, lower computational cost, and improved predictive performance. This has been previously demonstrated when classifying images into different categories using a public database named ImageNet^24,42^. This method is usually used to benchmark computer vision algorithms. In multiplexed image analysis, to allow precise interpretability with single-cell resolution, images were divided into a set of image patches, each containing one or two cells. To this end, we used our patch contrastive learning (PCL) strategy to embed pixel information into descriptive 256-dimensional representation vectors that gather both the size and shape of cells as well as marker expression and marker colocalization^43^. Specifically, a ResNet101-based convolutional neural network was trained with contrastive loss over 200 epochs and using a batch size of 80 image crops. Once the model was generated, it was used to create vector representations of all the images used in the study. Each multiplexed image was evenly divided into 20 × 20 × 7-pixel image patches with no overlap, and each patch was introduced into the PCL module to obtain a representation vector. Then, the spatial position of the patch in the image was stored. In this way, each TMA core was converted from a 2656 × 2656 × 7-pixel image into a list of 17,424 × 256 patches in the 256-representation space.

### Patch-graph generation

To allow graph neural networks to model the structure of the tissue and capture cellular neighborhoods as well as tissue areas, a graph of patches was created. An adjacency matrix was then created that contained the connectivity between patches, where each patch was connected to its four adjacent neighbors. If more than one image is available for a single patient, patches from distinct images were joined into a single graph. Compared to convolutional neural networks (CNNs) that input fixed-size images, GNNs are flexible when modeling the tumor microenvironment, allowing input from patients with different amounts of tissue available. In this case, patients have one or two tissue cores. From here on, we refer to patient tissue information instead of image information.

### NaroNet model

Our multilevel interpretable deep learning framework was used to generate a model to predict the risk of recurrence from multiplexed IF images of primary resection samples of low-grade, early-stage endometrial cancer. Our WSDL paradigm was used to handle large datasets and to capture the large heterogeneity seen in the microenvironment of these tumors. Patient classification occurred on the fixed, low-dimensional feature representation instead of the pixel space. In the feature space, NaroNet was trained to assign $L$ patches to elements from the tumor microenvironment at three levels of complexity:

- Local phenotype learning: This module allowed the assignment of individual patches to different categories (e.g., tumor cells {CK}, CD8 + T cells, etc.). To this end, patch representation vectors were forwarded through an 8-layer perceptron with skip connections, with the last layer being an assignment matrix $S_P \in {\mathbb R}^{L \times P}$, where $P$ consists of the number of local phenotypes learned. A softmax operation was applied to convert neuron activations into probability values so that $S_P$ row values sum to one representing the probability each patch had of belonging to each of the $P$ phenotypes.
- Cellular neighborhood learning: This module allowed the assignment of individual patches to different categories based on features of their neighbors (e.g., groups of tumor cells and tumor-infiltrating CD8 + T cells). To capture relationships between connected patches of a graph, a trainable weighted sum was applied so that each patch representation vector was updated with the features of their neighbors. This operation was performed $K$ times, or hops, to aggregate information from more spatially distant patches. As in local phenotype learning, the last layer of the GNN consisted of an assignment matrix $S_N \in {\mathbb R}^{L \times N}$, where $N$ is the number of neighborhoods, and a softmax operation was used to obtain probability values.
- Tissue area learning: This module allows the assignment of cellular neighborhoods to different categories based on its own features and other neighborhoods (e.g., an immune cold tumor, an immune hot tumor). To this end, a graph of neighborhoods was created, where nodes consist of cellular neighborhoods and edges represent connections. Similar to cellular neighborhood learning, neighborhood features were aggregated and assigned to ${{{\mathrm{A}}}}$ tissue areas with an assignment matrix $S_A \in {\mathbb R}^{N \times A}$.

From these patch assignment matrices $S_P,S_N,S_A$ NaroNet calculated a patient enrichment vector with a max-sum pooling operation:

1 $${{{\mathcal{P}}}} = \mathop {\sum }\limits_{1..L} \mathop {{\max }}\limits_{1..P} \left( {S_P} \right) \in {\mathbb R}^P$$

2 $${{{\mathcal{N}}}} = \mathop {\sum }\limits_{1..L} \mathop {{\max }}\limits_{1..N} \left( {S_N} \right) \in {\mathbb R}^N$$

3 $${{{\mathcal{A}}}} = \mathop {\sum }\limits_{1..L} \mathop {{\max }}\limits_{1..N} \left( {S_A} \right) \in {\mathbb R}^A$$

This intuitive trainable aggregation function allowed the network to obtain a patient vector that gathered how abundant each phenotype, neighborhood, and area was in the tissue. These abundance values were then used to calculate the final patient prediction score of recurrence. To this end, the patient enrichment vector $\left[ {{{{\mathcal{P}}}},{{{\mathcal{N}}}},{{{\mathcal{A}}}}} \right]$ was fed to a one-layer perceptron that outputs two final values, with one being the probability that patients will experience disease recurrence and the other the probability they will not experience recurrence. To obtain these probability values, a softmax operation was applied. The network parameters were updated using a cross-entropy function loss. The combination of trainable assignment matrices and max-sum pooling allowed the identification and annotation of tumor microenvironmental informative features from multiplexed images to predict the patient probability of disease recurrence. Further description of the method can be found in ref. ^16^.

### Hyperparameter tuning

To determine the configuration that provides the highest predictive performance, we incorporated an architecture search algorithm into the training phase. To this end, we use the Tune framework v1.0.0 with the ASHA search strategy to explore the search space^44,45^. We tested 400 model architectures for five different learning rates (1e-2, 1e-3, 5e-3, 1e-4, and 5e-4), for six weight decays (1e-3, 1e-4, 5e-4, 1e-5, 5e-5, and 1e-6), for three numbers of hops when learning neighborhoods (1, 2, and 3), for four neural network hidden dimensionalities (32, 64, 96, and 128), for six dropout rates (0.01, 0.05, 0.1, 0.2, 0.3, and 0.4), for four phenotypes (6, 9, 12, and 15), for four neighborhoods (7, 10, 13, and 16), and for four areas (8, 11, 14, and 17).

### Interpretability of the multilevel approach: local phenotypes, cellular neighborhoods, and areas

In addition to model predictions, NaroNet provided quantification of the tumor microenvironment at three levels of complexity: local phenotypes, cellular neighborhoods, and tissue areas. They can be interpreted using graphs and heatmaps.

At the local phenotype level, the 100 patches with the highest confidence levels were selected, and their marker-mean intensity was averaged and displayed in a heatmap. In this way, marker expression values can be compared within phenotypes. To measure colocalization between markers in phenotypes, the Spearman correlation was calculated. To this end, a Spearman value was calculated between pairs of markers for each patch and then averaged and displayed in a heatmap. To measure nucleus morphology parameters, Stardist^25^ was used on the DAPI channels for each patch, and segmentation masks were introduced in Morpholibj to extract features (i.e., nucleus area, cell density, and nucleus circularity)^26^.

Next, cellular neighborhoods were defined as interactions, or spatial affinities, between local phenotypes. As patches were assigned to a neighborhood based on their own features as well as their k-hop adjacent ones, a k = 2 hop-based neighborhood was composed of 12 patches. Then, for each neighborhood, we calculated the local phenotypes to which adjacent patches were assigned. This was exhaustively done for all neighborhoods in all images from the patient cohort. Once count values were computed, cellular neighborhood composition was visualized in a heatmap by performing a z-scored normalization, showing all the local phenotypes that make up the neighborhoods.

Next, tissue areas were defined as interactions, or spatial affinities, between cellular neighborhoods. As areas were calculated from a graph of neighborhoods, each of the neighborhoods was assigned to one area or another. Images were exhaustively evaluated to obtain the neighborhood assignment to areas. These counts were area-wise *z*-score normalized and visualized in a heatmap.

Finally, to investigate the association between local phenotypes, cellular neighborhoods, and tissue areas with clinical characteristics and molecular subtypes, we compared the relative abundance of each of these features with different clinical, pathological, and molecular characteristics of the tumors. Associations with FIGO stage and age were analyzed by Spearman correlation. The other variables were analyzed by a two-sided *t*-test. All *p* values were penalized using False Discovery Reach.

### Visualization of images

To interpret the local phenotypes, cellular neighborhoods and the tissue area composition of the different regions in a given tissue image, we computed and saved the normalized assignment scores (after they were converted to a probability distribution by applying the softmax function) for all the patches extracted from a tissue image. The normalized assignment scores for each element from the tumor microenvironment (local phenotypes, cellular neighborhoods, and tissue areas) were converted to an RGB scale using a diverging color map and displayed on top of their respective spatial locations in the image to visually identify and interpret microenvironmentally distinct regions that were associated with recurrence. Patches with high confidence values are displayed in yellow, and patches with low-confidence values are displayed in black (Fig. 6, top right images).

### Molecular subtype characterization

Mismatch repair protein (MMRP) status and p53 expression pattern were determined by IHC as described previously^46,47^. Briefly, immunohistochemistry was performed on TMA sections using the following primary antibodies: MLH1 (prediluted, ES05, Dako-Agilent), PMS2 (prediluted, EP51, Dako-Agilent), MSH2 (prediluted, FE11, Dako-Agilent), MSH6 (prediluted, EP49, Dako-Agilent), and p53 (prediluted, DO-7, Dako-Agilent). For mismatch repair proteins, a partial or complete absence of nuclear expression of any of the markers was considered abnormal. For p53 pattern determination, either diffuse intense nuclear expression, absent nuclear expression, or cytoplasmic staining was considered abnormal. Mutations in the POLE exonuclease domain (exons 9, 11, 13, and 14) were detected by Sanger sequencing as described previously in ref. ^46^.

### Reporting summary

Further information on research design is available in the Nature Research Reporting Summary linked to this article.

## Supplementary information

Supplementary Information

REPORTING SUMMARY

Supplementary Data

## Data Availability

De-identified study data may be made available at publication upon request to the corresponding author. Data sharing will only be available for academic research instead of commercial use or other objectives. A data use agreement and institutional review board approval will be required as appropriate.

## Code availability

All code was implemented in Python using PyTorch. All code and scripts are available at: https://github.com/djimenezsanchez/NaroNet.
