# Supplementary material for: Weakly supervised deep learning to predict recurrence in low-grade endometrial cancer from multiplexed immunofluorescence images
Source: NPJ Digit Med. 2023 Mar 23;6:48. doi: 10.1038/s41746-023-00795-x (PMC10036616; doi:10.1038/s41746-023-00795-x)
Supplement: Supplementary file 1 — Supplementary Information [file 41746_2023_795_MOESM1_ESM.pdf]

Supplementary Information

Weakly supervised deep learning to predict recurrence in low-grade endometrial cancer from multiplexed immunofluorescence images

Daniel Jiménez-Sánchez[1,2], Álvaro López-Janeiro[2,3], María Villalba-Esparza[2,4], Mikel Ariz[1,4], Ece Kadioglu[5], Ivan Masetto[6], Virginie Goubert[6], Maria D. Lozano[2,4,7], Ignacio Melero[4,7,8,9], David Hardisson[3,7,10,11], Carlos Ortiz-de-Solórzano[1,4,7], Carlos E de Andrea[2,4,7,*]

1. Program of Solid Tumors and Biomarkers, Center for Applied Medical Research (CIMA), University of Navarra, Pamplona, Spain
2. Department of Pathology, Clínica Universidad de Navarra, Pamplona, Spain
3. Department of Pathology, Hospital Universitario La Paz, IdiPAZ, Madrid, Spain
4. Navarra Institute for Health Research (IdISNA), Pamplona, Spain
5. Lunaphore Technologies SA, Tolochenaz, Switzerland
6. Akoya Biosciences, Marlborough, MA, USA
7. Center for Biomedical Research in the Cancer Network (CIBERONC), Madrid, Spain
8. Department of Immunology and Immunotherapy, Clínica Universidad de Navarra, Pamplona, Spain.
9. Program of Immunology and Immunotherapy, Center for Applied Medical Research (CIMA), University of Navarra, Pamplona, Spain
10. Molecular Pathology and Therapeutic Targets Group, La Paz University Hospital, IdiPAZ, Madrid, Spain
11. Faculty of Medicine, Universidad Autónoma de Madrid, Madrid, Spain

# Corresponding author
Carlos E de Andrea, MD, PhD
Department of Pathology
Clínica Universidad de Navarra
Av. de Pío XII 36
31008, Pamplona, Navarra
Spain
Phone: +34 948 25 54 00
Email: ceandrea@unav.es

Keywords: endometrial cancer, low-grade, early-stage, recurrence, prediction, immune-microenvironment, multiplex quantitative immunofluorescence, weakly-supervised, deep learning

Supplementary Figure 1

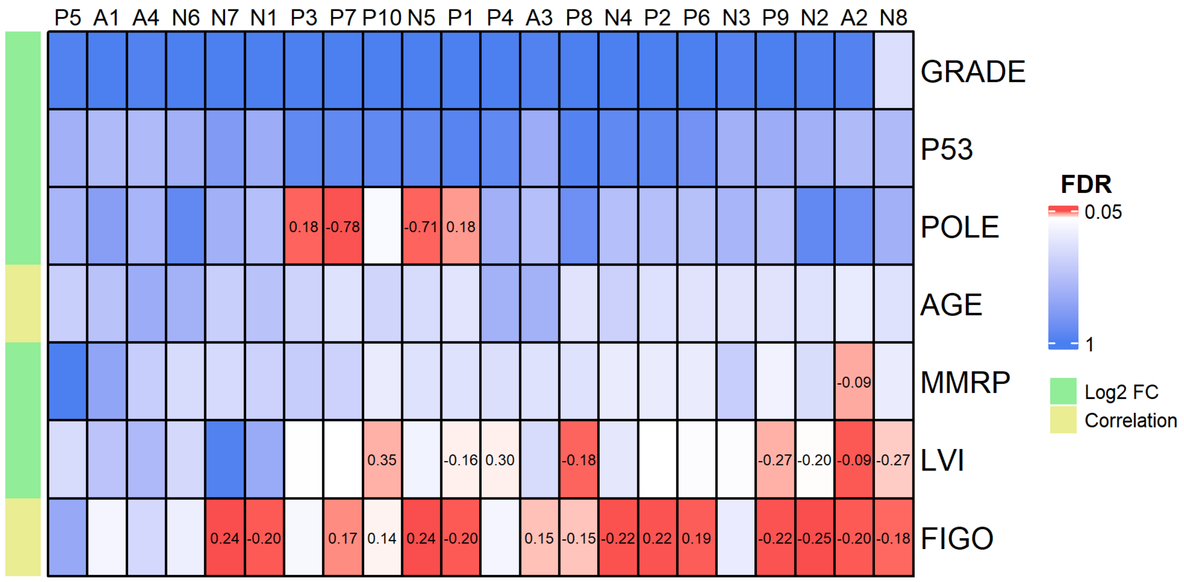

Supplementary Fig. 1: Heatmap showing the false discovery reach (FDR) corrected *p* values of associations between local phenotypes, cellular neighborhoods, and tissue areas with clinicopathologic and molecular information.

Grade includes stages I and II. p53 protein expression was detected by immunohistochemistry. POLE represents tumors with mutations in the POLE exonuclease domain (exons 9, 11, 13, and 14). MMRP includes associations with mismatch repair protein (MMRP) deficient tumors. LVI refers to lymphovascular invasion. FIGO ranges from stage G1 through G2. Two-sided Student t test were used for all variables except age and FIGO stage. For these two variables ranked spearman correlation test was applied. Log2 Fold change and Spearman correlation index are shown for significantly associated features.

Supplementary Figure 2

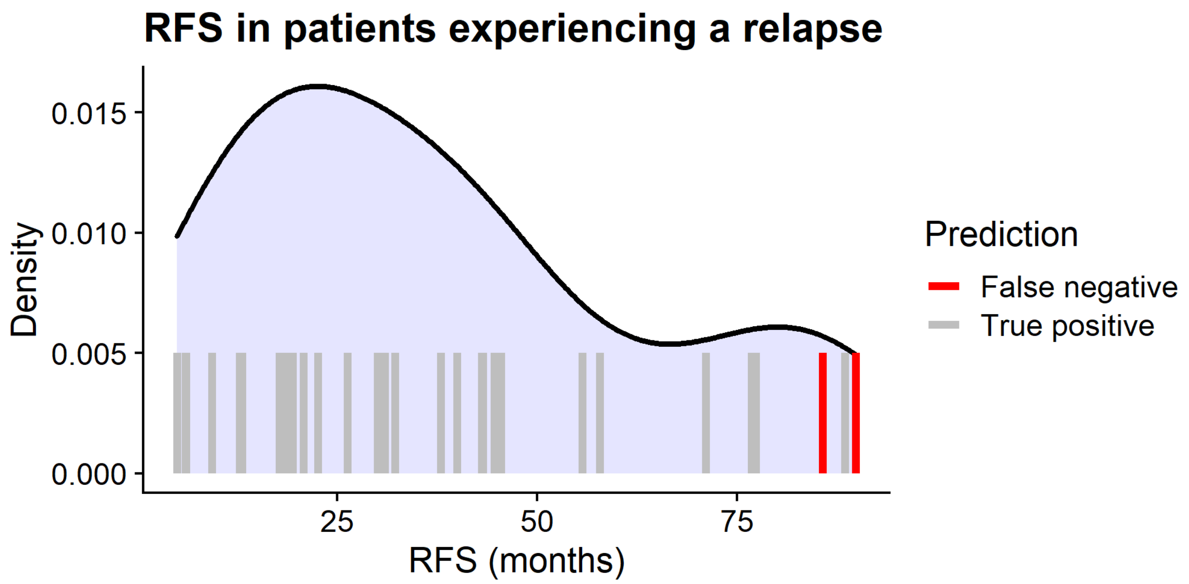

Supplementary Fig. 2: Relapse pattern. Smoothened histogram showing the distribution of relapse-free survival (RFS) in patients with tumor recurrence. Patients are indicated as colored bars. The two patients incorrectly misclassified as non-recurrence developed late endometrial cancer recurrence at 86 and 90 months after resection (>percentile 95).

Supplementary Table 1: Clinicopathological and molecular characteristics separately for patients with recurrent tumors and those with non-recurrent tumors.

**Clinical and pathological features**	**All tumors (n = 250)**	**No Relapse (n = 218)**	**Relapse (n = 32)**
Age (y, mean, p25-75)	64.5 (57 – 73)	64.1 (56 – 73)	66.9 (60 – 74)
FIGO stage	IA = 171 (68.4%)<br>IB = 66 (26.4%)<br>II = 13 (5.2%)	IA = 160 (73.4%)<br>IB = 49 (22.5%)<br>II = 9 (4.1%)	IA = 11 (34.4%)<br>IB = 17 (53.1%)<br>II = 4 (12.5%)
FIGO grade	G1 = 195 (78%)<br>G2 = 55 (22%)	G1 = 176 (80.7%)<br>G2 = 42 (19.3%)	G1 = 19 (59.4%)<br>G2 = 13 (40.6%)
Presence of lymphovascular invasion	Absent = 201 (80.4%)<br>Present = 49 (19.6%)	Absent = 185 (84.9%)<br>Present = 33 (15.1%)	Absent = 16 (50%)<br>Present = 16 (50%)
Mismatch repair protein status	Proficient = 175 (70%)<br>Deficient = 45 (18%)<br>Not Available = 30 (12%)	Proficient = 159 (72.9%)<br>Deficient = 30 (13.8%)<br>Not Available = 29 (13.3%)	Proficient = 16 (50%)<br>Deficient = 15 (46.9%)<br>Not Available = 1 (3.1%)
POLE (exons 9-11-13-14) mutation status	Wild Type = 136 (54.4%)<br>Mutated = 8 (3.2%)<br>Not Available = 106 (42.4%)	Wild Type = 121 (55.5%)<br>Mutated = 8 (3.7%)<br>Not Available = 89 (40.8%)	Wild Type = 15 (46.9%)<br>Mutated = 0<br>Not Available = 17 (53.1%)
p53 Immunohistochemistry	Wild Type Pattern = 242 (96.8%)<br>Aberrant Pattern = 7 (2.8%)<br>Inconclusive = 1 (0.4%)	Wild Type Pattern = 213 (97.7%)<br>Aberrant Pattern = 4 (1.8%)<br>Inconclusive = 1 (0.5%)	Wild Type Pattern = 29 (90.6%)<br>Aberrant Pattern = 3 (9.4%)<br>Inconclusive = 0
Follow up (m, median, p25-75)	120 (77.3 – 120)	120 (93.2 – 120)	30.9 (18.3 – 50.5)
Adjuvant radiotherapy	No radiotherapy = 156 (62.4%)<br>EBRT = 3 (1.2%)<br>VBT = 25 (10%)<br>EBRT + VBT = 36 (14.4%)<br>Not Available = 30 (12%)	No radiotherapy = 145 (66.5%)<br>EBRT = 3 (1.4%)<br>VBT = 19 (8.7%)<br>EBRT + VBT = 25 (14.5%)<br>Not Available = 26 (11.9%)	No radiotherapy = 11 (34.4%)<br>EBRT = 0<br>VBT = 6 (18.8%)<br>EBRT + VBT = 11 (34.4%)<br>Not Available = 4 (12.5%)

y = years, m = months, MMRPd = Mismatch Repair Proteins Deficient, EBRT = External Beam Radiation Therapy, VBT = Vault Brachytherapy.

Supplementary Table 2: Hyperparameters chosen by the architecture search algorithm.

Parameter name	Value
Learning rate	0.01
Weight decay	1e-6
Number of hops	2
Hidden layers dimensions	64
Dropout rate	40%
Number of local phenotypes	10
Number of cellular neighborhoods	8
Number of tissue areas	4

Supplementary Table 3: NaroNet recurrence prediction values for all patients.

Subject Name	NaroNet recurrence prediction	NaroNet final decision	Long-term follow-up
A1	[0.00390279 0.99609727]	No recurrence	No recurrence
A2	[9.9957675e-01 4.2324958e-04]	No recurrence	No recurrence
A3	[0.9976673 0.00233269]	No recurrence	No recurrence
A4	[0.98818284 0.01181717]	No recurrence	No recurrence
A5	[3.2034342e-05 9.9996793e-01]	Recurrence	Recurrence
A6	[0.9523875 0.04761249]	No recurrence	No recurrence
A7	[0.8293646 0.17063543]	No recurrence	No recurrence
A8	[9.9996340e-01 3.6616035e-05]	No recurrence	No recurrence
A9	[0.01077238 0.98922765]	No recurrence	No recurrence
A10	[0.14275923 0.85724074]	No recurrence	No recurrence
A11	[4.6337118e-05 9.9995363e-01]	No recurrence	No recurrence
A12	[0.97792995 0.02207003]	No recurrence	No recurrence
A13	[0.8517386 0.14826147]	Recurrence	Recurrence
A14	[0.23596905 0.764031]	No recurrence	No recurrence
A15	[0.9944687 0.00553136]	No recurrence	No recurrence
A16	[0.00695104 0.9930489]	No recurrence	No recurrence
A17	[0.99857175 0.00142821]	No recurrence	No recurrence
A18	[0.9984158 0.0015842]	No recurrence	No recurrence
A19	[0.7851653 0.21483469]	No recurrence	No recurrence
A20	[3.3142234e-04 9.9966860e-01]	No recurrence	No recurrence
A21	[0.9255238 0.07447616]	No recurrence	No recurrence
A22	[3.2625903e-04 9.9967372e-01]	Recurrence	Recurrence
A23	[0.01935928 0.98064077]	No recurrence	No recurrence
A24	[0.01006834 0.9899317]	Recurrence	Recurrence
A25	[0.00186207 0.99813795]	No recurrence	No recurrence
A26	[0.9187062 0.08129384]	No recurrence	No recurrence
A27	[9.9999821e-01 1.8250322e-06]	No recurrence	No recurrence
A28	[0.96712923 0.03287073]	No recurrence	No recurrence
A29	[1.5204791e-06 9.9999845e-01]	Recurrence	Recurrence

A30	[0.97271574 0.0272843]	No recurrence	No recurrence
A31	[0.9947418 0.00525823]	No recurrence	No recurrence
A32	[0.99860185 0.00139816]	No recurrence	No recurrence
A33	[0.9595118 0.04048818]	No recurrence	No recurrence
A34	[0.00152402 0.99847597]	No recurrence	No recurrence
A35	[9.990396e-01 9.603648e-04]	No recurrence	No recurrence
A36	[0.80584115 0.19415891]	No recurrence	No recurrence
A37	[0.9350018 0.06499814]	No recurrence	No recurrence
A38	[0.9978381 0.0021619]	No recurrence	No recurrence
A39	[0.9818827 0.01811732]	No recurrence	No recurrence
A40	[7.0347662e-05 9.9992967e-01]	Recurrence	Recurrence
A41	[0.95028925 0.0497107]	No recurrence	No recurrence
A42	[2.2388998e-04 9.9977607e-01]	Recurrence	Recurrence
A43	[0.0441306 0.9558694]	No recurrence	Recurrence
A44	[0.876117 0.12388302]	No recurrence	No recurrence
A45	[0.6612539 0.33874607]	No recurrence	No recurrence
A46	[0.6368655 0.36313447]	No recurrence	No recurrence
A47	[0.9357962 0.06420378]	No recurrence	No recurrence
A48	[9.9991894e-01 8.1112223e-05]	No recurrence	No recurrence
A49	[0.8789791 0.12102093]	No recurrence	No recurrence
A50	[0.62946355 0.37053648]	No recurrence	No recurrence
A51	[0.05579726 0.9442028]	No recurrence	No recurrence
A52	[9.9914885e-01 8.5115427e-04]	No recurrence	No recurrence
A53	[9.300102e-09 1.000000e+00]	Recurrence	Recurrence
A54	[0.14206062 0.8579394]	No recurrence	No recurrence
A55	[0.9244833 0.07551665]	No recurrence	No recurrence
A56	[9.9980968e-01 1.9030515e-04]	No recurrence	No recurrence
A57	[9.9966431e-01 3.3565043e-04]	No recurrence	No recurrence
A58	[1.12155256e-04 9.998878e-01]	Recurrence	Recurrence
A59	[0.7001692 0.29983082]	Recurrence	Recurrence
A60	[0.99816304 0.00183698]	No recurrence	No recurrence
A61	[0.9005814 0.09941858]	No recurrence	No recurrence
A62	[0.15047175 0.84952825]	No recurrence	No recurrence

A63	[0.9672706 0.03272933]	No recurrence	No recurrence
A64	[9.9976820e-01 2.3181457e-04]	No recurrence	No recurrence
A65	[1.381285e-08 1.00000000e+00]	Recurrence	Recurrence
A66	[5.1736023e-04 9.9948263e-01]	No recurrence	No recurrence
A67	[0.19741857 0.80258137]	No recurrence	No recurrence
A68	[0.08105057 0.9189494]	No recurrence	No recurrence
A69	[0.9871308 0.01286917]	No recurrence	No recurrence
A70	[1.7043050e-04 9.9982953e-01]	No recurrence	No recurrence
A71	[9.9941516e-01 5.8486534e-04]	No recurrence	No recurrence
A72	[0.9917591 0.00824091]	No recurrence	No recurrence
A73	[0.9977888 0.00221123]	No recurrence	No recurrence
A74	[4.8392956e-04 9.9951613e-01]	No recurrence	No recurrence
A75	[0.85568625 0.14431374]	No recurrence	No recurrence
A76	[9.9927956e-01 7.2045339e-04]	No recurrence	No recurrence
A77	[8.417409e-08 9.999999e-01]	Recurrence	Recurrence
A78	[0.5432023 0.45679772]	No recurrence	No recurrence
A79	[0.9978447 0.00215535]	No recurrence	No recurrence
A80	[0.93730396 0.06269608]	No recurrence	No recurrence
A81	[0.00429444 0.99570554]	No recurrence	No recurrence
A82	[0.9622934 0.03770665]	No recurrence	No recurrence
A83	[0.16042216 0.83957785]	No recurrence	No recurrence
A84	[9.9937636e-01 6.2363583e-04]	No recurrence	No recurrence
A85	[0.85622513 0.14377488]	No recurrence	No recurrence
A86	[0.9926756 0.00732444]	No recurrence	No recurrence
A87	[4.616849e-07 9.999995e-01]	Recurrence	Recurrence
A88	[0.01220752 0.98779243]	Recurrence	Recurrence
A89	[0.8342809 0.16571906]	No recurrence	No recurrence
A90	[0.96009946 0.0399005]	No recurrence	No recurrence
A91	[0.99869555 0.00130453]	No recurrence	No recurrence
A92	[0.90103686 0.09896316]	No recurrence	No recurrence
A93	[0.9989262 0.00107381]	No recurrence	No recurrence
A94	[4.9756813e-06 9.9999499e-01]	Recurrence	Recurrence
A95	[0.48654717 0.5134529]	No recurrence	No recurrence

A96	[0.55582017 0.4441798]	No recurrence	No recurrence
A97	[0.9936813 0.00631872]	No recurrence	No recurrence
A98	[0.9921257 0.00787437]	No recurrence	No recurrence
A99	[0.97497195 0.02502802]	No recurrence	No recurrence
A100	[0.01125668 0.9887433]	No recurrence	No recurrence
A101	[0.00412094 0.99587905]	No recurrence	No recurrence
A102	[0.9750794 0.02492064]	No recurrence	No recurrence
A103	[0.3252287 0.6747713]	No recurrence	No recurrence
A104	[1.7009336e-08 1.0000000e+00]	Recurrence	Recurrence
A105	[0.9863884 0.01361161]	No recurrence	No recurrence
A106	[0.5963018 0.40369815]	No recurrence	No recurrence
A107	[0.93947417 0.06052589]	No recurrence	No recurrence
A108	[0.90983653 0.09016354]	No recurrence	No recurrence
A109	[0.9669129 0.03308706]	No recurrence	No recurrence
A110	[0.9893911 0.01060884]	No recurrence	No recurrence
A111	[9.9985242e-01 1.4757986e-04]	No recurrence	No recurrence
A112	[0.00132349 0.9986765]	Recurrence	Recurrence
A113	[0.98241156 0.01758846]	No recurrence	No recurrence
A114	[0.99862826 0.00137172]	No recurrence	No recurrence
A115	[0.9030789 0.09692111]	No recurrence	No recurrence
A116	[0.11612775 0.8838723]	No recurrence	No recurrence
A117	[1.8421691e-06 9.9999821e-01]	Recurrence	Recurrence
A118	[0.99884355 0.00115637]	No recurrence	No recurrence
A119	[0.99780864 0.00219141]	No recurrence	No recurrence
A120	[0.88437843 0.11562157]	No recurrence	No recurrence
A121	[2.2384513e-07 9.9999976e-01]	Recurrence	Recurrence
A122	[0.7591261 0.24087381]	No recurrence	No recurrence
A123	[0.00138788 0.99861205]	No recurrence	No recurrence
A124	[0.2684125 0.7315875]	No recurrence	No recurrence
A125	[9.9971288e-01 2.8705268e-04]	No recurrence	No recurrence
A126	[0.8913015 0.10869852]	No recurrence	No recurrence
A127	[0.9720928 0.02790712]	No recurrence	No recurrence
A128	[0.03302814 0.96697193]	No recurrence	No recurrence

A129	[0.5881702 0.41182983]	No recurrence	No recurrence
A130	[0.9794057 0.02059423]	No recurrence	No recurrence
A131	[0.9725719 0.02742803]	Recurrence	No recurrence
A132	[9.729766e-06 9.999902e-01]	No recurrence	No recurrence
A133	[0.9981852 0.00181471]	No recurrence	No recurrence
A134	[8.1658596e-05 9.9991834e-01]	No recurrence	No recurrence
A135	[0.99861467 0.00138527]	No recurrence	No recurrence
A136	[0.99867404 0.00132599]	No recurrence	No recurrence
A137	[0.06720287 0.93279713]	Recurrence	No recurrence
A138	[3.9734363e-04 9.9960274e-01]	No recurrence	No recurrence
A139	[0.5806955 0.4193045]	Recurrence	No recurrence
A140	[0.04647445 0.9535256]	No recurrence	No recurrence
A141	[0.7787156 0.22128439]	No recurrence	No recurrence
A142	[0.04224619 0.9577538]	No recurrence	No recurrence
A143	[6.7338835e-05 9.9993265e-01]	No recurrence	No recurrence
A144	[0.6376501 0.36234996]	No recurrence	No recurrence
A145	[0.8041793 0.19582073]	No recurrence	No recurrence
A146	[0.8042569 0.19574304]	No recurrence	No recurrence
A147	[3.6152503e-06 9.9999642e-01]	Recurrence	Recurrence
A148	[0.12598684 0.8740132]	No recurrence	No recurrence
A149	[0.9886296 0.01137038]	No recurrence	No recurrence
A150	[0.48715985 0.5128402]	No recurrence	No recurrence
A151	[2.6515067e-06 9.9999738e-01]	Recurrence	Recurrence
A152	[0.9848081 0.01519191]	No recurrence	No recurrence
A153	[0.66145635 0.33854368]	No recurrence	No recurrence
A154	[0.98427653 0.0157235]	No recurrence	No recurrence
A155	[0.5723915 0.42760855]	No recurrence	No recurrence
A156	[0.99454063 0.00545939]	No recurrence	No recurrence
A157	[9.9994290e-01 5.7082707e-05]	No recurrence	No recurrence
A158	[0.98678684 0.01321309]	No recurrence	No recurrence
A159	[0.13260004 0.8674]	No recurrence	No recurrence
A160	[0.09022766 0.9097724]	No recurrence	No recurrence
A161	[0.880439 0.11956104]	No recurrence	No recurrence

A162	[0.78084075 0.21915923]	No recurrence	No recurrence
A163	[0.99823415 0.00176581]	No recurrence	No recurrence
A164	[0.04724593 0.9527541]	No recurrence	No recurrence
A165	[7.9786964e-04 9.9920219e-01]	No recurrence	No recurrence
A166	[0.77464175 0.22535825]	No recurrence	No recurrence
A167	[1.106557e-04 9.998894e-01]	Recurrence	Recurrence
A168	[0.4863244 0.5136756]	No recurrence	No recurrence
A169	[0.9714334 0.02856664]	No recurrence	No recurrence
A170	[0.05030447 0.94969547]	No recurrence	No recurrence
A171	[8.3997620e-06 9.9999166e-01]	Recurrence	Recurrence
A172	[0.97451586 0.0254842]	No recurrence	Recurrence
A173	[2.6280698e-04 9.9973720e-01]	Recurrence	Recurrence
A174	[0.01238715 0.9876129]	No recurrence	No recurrence
A175	[0.82945 0.17055002]	No recurrence	No recurrence
A176	[0.8089137 0.19108637]	No recurrence	No recurrence
A177	[0.9637636 0.03623641]	No recurrence	No recurrence
A178	[0.37152737 0.6284727]	No recurrence	No recurrence
A179	[0.82341594 0.17658412]	No recurrence	No recurrence
A180	[0.19527094 0.8047291]	No recurrence	No recurrence
A181	[9.996209e-01 3.790865e-04]	No recurrence	No recurrence
A182	[9.9996543e-01 3.4517132e-05]	No recurrence	No recurrence
A183	[0.99084926 0.00915081]	No recurrence	No recurrence
A184	[0.67940015 0.32059988]	No recurrence	No recurrence
A185	[9.9999797e-01 2.0820187e-06]	No recurrence	No recurrence
A186	[0.00987177 0.9901282]	No recurrence	No recurrence
A187	[0.03037629 0.9696237]	No recurrence	No recurrence
A188	[4.8138489e-04 9.9951863e-01]	Recurrence	Recurrence
A189	[0.52934307 0.47065687]	No recurrence	No recurrence
A190	[0.9470525 0.05294752]	No recurrence	No recurrence
A191	[0.00497545 0.9950245]	No recurrence	No recurrence
A192	[0.95752233 0.04247766]	No recurrence	No recurrence
A193	[0.8144476 0.18555245]	Recurrence	Recurrence
A194	[0.9796856 0.02031445]	No recurrence	No recurrence

A195	[0.00502329 0.9949767]	No recurrence	No recurrence
A196	[0.9920806 0.00791935]	No recurrence	No recurrence
A197	[2.9219568e-04 9.9970776e-01]	Recurrence	No recurrence
A198	[0.9809202 0.01907973]	No recurrence	No recurrence
A199	[9.999064e-01 9.360976e-05]	No recurrence	No recurrence
A200	[0.83339405 0.16660587]	No recurrence	No recurrence
A201	[0.1151382 0.88486177]	No recurrence	No recurrence
A202	[0.91637033 0.08362968]	No recurrence	No recurrence
A203	[0.4735041 0.5264959]	No recurrence	No recurrence
A204	[0.42568496 0.5743151]	No recurrence	No recurrence
A205	[0.00610865 0.99389136]	No recurrence	No recurrence
A206	[0.01016998 0.98982996]	No recurrence	No recurrence
A207	[0.9873225 0.01267743]	No recurrence	No recurrence
A208	[0.02702397 0.972976]	No recurrence	No recurrence
A209	[0.6326517 0.3673483]	No recurrence	No recurrence
A210	[0.9979243 0.00207564]	Recurrence	No recurrence
A211	[0.9427382 0.05726184]	No recurrence	No recurrence
A212	[0.96368986 0.03631013]	No recurrence	No recurrence
A213	[0.9441999 0.05580012]	No recurrence	No recurrence
A214	[0.9315038 0.06849623]	No recurrence	No recurrence
A215	[0.8181 0.18190001]	No recurrence	No recurrence
A216	[0.82060945 0.1793905]	No recurrence	No recurrence
A217	[0.9714451 0.02855494]	No recurrence	No recurrence
A218	[0.97714305 0.02285689]	No recurrence	No recurrence
A219	[5.4975029e-05 9.9994504e-01]	No recurrence	No recurrence
A220	[0.98936266 0.01063737]	No recurrence	No recurrence
A221	[0.12199204 0.878008]	Recurrence	No recurrence
A222	[1.9382597e-04 9.9980623e-01]	No recurrence	No recurrence
A223	[1.12866015e-04 9.998871e-01]	Recurrence	Recurrence
A224	[0.8379518 0.16204825]	No recurrence	No recurrence
A225	[0.90429425 0.09570571]	No recurrence	No recurrence
A226	[0.6577464 0.34225363]	No recurrence	No recurrence
A227	[1.7169036e-04 9.9982834e-01]	No recurrence	No recurrence

A228	[0.9989507 0.00104925]	No recurrence	No recurrence
A229	[0.00732351 0.9926765]	No recurrence	No recurrence
A230	[0.9389658 0.0610342]	No recurrence	No recurrence
A231	[0.08568032 0.9143197]	No recurrence	No recurrence
A232	[0.03219355 0.9678065]	No recurrence	No recurrence
A233	[0.97297746 0.02702257]	No recurrence	No recurrence
A234	[0.72571164 0.27428836]	No recurrence	No recurrence
A235	[0.55752766 0.4424723]	No recurrence	No recurrence
A236	[0.86918557 0.1308144]	No recurrence	No recurrence
A237	[0.8307884 0.16921166]	No recurrence	No recurrence
A238	[0.9512504 0.0487496]	No recurrence	No recurrence
A239	[0.4230967 0.5769033]	Recurrence	Recurrence
A240	[0.5085587 0.4914413]	No recurrence	No recurrence
A241	[0.25637662 0.7436233]	No recurrence	No recurrence
A242	[9.9985468e-01 1.4525783e-04]	No recurrence	No recurrence
A243	[0.03056257 0.9694374]	No recurrence	No recurrence
A244	[0.09456389 0.90543604]	Recurrence	Recurrence
A245	[0.36615464 0.6338453]	No recurrence	No recurrence
A246	[0.02308244 0.9769175]	No recurrence	No recurrence
A247	[0.24319491 0.7568051]	No recurrence	No recurrence
A248	[0.9911793 0.00882068]	No recurrence	No recurrence
A249	[4.8247131e-04 9.9951756e-01]	Recurrence	Recurrence
A250	[0.9983923 0.00160773]	No recurrence	No recurrence

Supplementary Table 4: Relative abundances of local phenotypes, cellular neighborhoods, and tissue areas for each patient. These abundances were used to predict tumor recurrence. *See additional spreadsheet uploaded in a separate file*.
