# Supplementary material for: Weakly supervised deep learning to predict recurrence in low-grade endometrial cancer from multiplexed immunofluorescence images
Source: NPJ Digit Med. 2023 Mar 23;6:48. doi: 10.1038/s41746-023-00795-x (PMC10036616; doi:10.1038/s41746-023-00795-x)
Supplement: Supplementary file 2 — REPORTING SUMMARY [file 41746_2023_795_MOESM2_ESM.pdf]

nature portfolio

Corresponding author(s): Carlos E de Andrea
Last updated by author(s): 2022/12/11

Reporting Summary

Nature Portfolio wishes to improve the reproducibility of the work that we publish. This form provides structure for consistency and transparency in reporting. For further information on Nature Portfolio policies, see our Editorial Policies and the Editorial Policy Checklist.

Statistics

For all statistical analyses, confirm that the following items are present in the figure legend, table legend, main text, or Methods section.

n/a	Confirmed	
☐	☒	The exact sample size (*n*) for each experimental group/condition, given as a discrete number and unit of measurement
☐	☒	A statement on whether measurements were taken from distinct samples or whether the same sample was measured repeatedly
☐	☒	The statistical test(s) used AND whether they are one- or two-sided<br>*Only common tests should be described solely by name; describe more complex techniques in the Methods section.*
☐	☒	A description of all covariates tested
☐	☒	A description of any assumptions or corrections, such as tests of normality and adjustment for multiple comparisons
☐	☒	A full description of the statistical parameters including central tendency (e.g. means) or other basic estimates (e.g. regression coefficient) AND variation (e.g. standard deviation) or associated estimates of uncertainty (e.g. confidence intervals)
☐	☒	For null hypothesis testing, the test statistic (e.g. *F*, *t*, *r*) with confidence intervals, effect sizes, degrees of freedom and *P* value noted<br>*Give P values as exact values whenever suitable.*
☒	☐	For Bayesian analysis, information on the choice of priors and Markov chain Monte Carlo settings
☐	☒	For hierarchical and complex designs, identification of the appropriate level for tests and full reporting of outcomes
☐	☒	Estimates of effect sizes (e.g. Cohen's *d*, Pearson's *r*), indicating how they were calculated

Our web collection on statistics for biologists contains articles on many of the points above.

Software and code

Policy information about availability of computer code

Data collection	Not appropriate since our data are retrospective samples seen at the Pathology Department of the University Hospital La Paz (Madrid, Spain)
Data analysis	All code was implemented in Python using PyTorch. For imaging pre-processing, ImageJ software was used.

For manuscripts utilizing custom algorithms or software that are central to the research but not yet described in published literature, software must be made available to editors and reviewers. We strongly encourage code deposition in a community repository (e.g. GitHub). See the Nature Portfolio guidelines for submitting code & software for further information.

Data

Policy information about availability of data

All manuscripts must include a data availability statement. This statement should provide the following information, where applicable:

- Accession codes, unique identifiers, or web links for publicly available datasets
- A description of any restrictions on data availability
- For clinical datasets or third party data, please ensure that the statement adheres to our policy

De-identified study data may be made available at publication upon request to the corresponding author. Data sharing will only be available for academic research, instead of commercial use or other objectives. A data use agreement and institutional review board approval will be required as appropriate.

Human research participants

Policy information about studies involving human research participants and Sex and Gender in Research.

Reporting on sex and gender	All patients were female with low-grade, early-stage endometrial cancer.
Population characteristics	The mean age of the patients was 64.5 years.
Recruitment	Retrospective study. Samples collected at the Pathology Department of the University Hospital La Paz (Madrid, Spain)
Ethics oversight	All tissues were used after approval from the University Hospital La Paz Human Research Committee, protocol number: HULP: PI-3108.

Note that full information on the approval of the study protocol must also be provided in the manuscript.

Field-specific reporting

Please select the one below that is the best fit for your research. If you are not sure, read the appropriate sections before making your selection.

☒ Life sciences ☐ Behavioural & social sciences ☐ Ecological, evolutionary & environmental sciences

For a reference copy of the document with all sections, see nature.com/documents/nr-reporting-summary-flat.pdf

Life sciences study design

All studies must disclose on these points even when the disclosure is negative.

Sample size	250 patients with low-grade endometrioid carcinomas seen at the Pathology Department of the University Hospital La Paz (Madrid, Spain)
Data exclusions	All patients that did not met the following inclusion criteria were excluded: (i) surgical treatment and long-term follow-up undertaken at the University Hospital La Paz; (ii) all tumors were low-grade (G1 or G2) endometrioid carcinomas, stages I and II, according to the 2009 International Federation of Gynecology and Obstetrics (FIGO) classification; (iii) all tumors had wild-type p53 protein detected by immunohistochemistry; (iv) patients did not undergo neoadjuvant/adjuvant systemic treatment or immunotherapy; and (v) because most tumors recur in the first 3 years after diagnosis, all patients had a minimum follow-up period of 3 years.
Replication	A 10-fold cross-validation strategy was used to measure the classification performance for predicting tumor recurrence. In each fold, 225 patients (90%) were used to train the model, whereas the remaining 25 patients (10%) were used to test the model.
Randomization	NA
Blinding	NA

Reporting for specific materials, systems and methods

We require information from authors about some types of materials, experimental systems and methods used in many studies. Here, indicate whether each material, system or method listed is relevant to your study. If you are not sure if a list item applies to your research, read the appropriate section before selecting a response.

Materials & experimental systems

n/a	Involved in the study	
☐	☒	Antibodies
☒	☐	Eukaryotic cell lines
☒	☐	Palaeontology and archaeology
☒	☐	Animals and other organisms
☐	☒	Clinical data
☒	☐	Dual use research of concern

Methods

n/a	Involved in the study	
☒	☐	ChIP-seq
☒	☐	Flow cytometry
☒	☐	MRI-based neuroimaging

Antibodies

Antibodies used	Cytokeratin (pan-CK) (1:150 clone AE1/AE3, Novus Biologicals), CD8 (1:150, clone 4B11, Bio-rad), CD68 (1:75, clone PG-M1, Dako-Agilent), FOXP3 (1:50, clone 236A/E7, Abcam), PD-1(1:300, ERP4877, Abcam), and PD-L1 (1:300, clone E1L3N, Cell Signaling).

Validation	Singleplex assays were used as the gold standard for cell antigen visualization. A singleplex versus multiplexed comparison for each antibody was performed to validate the staining patterns and distribution.

Clinical data

Policy information about clinical studies

All manuscripts should comply with the ICMJE guidelines for publication of clinical research and a completed CONSORT checklist must be included with all submissions.

Clinical trial registration	NA
Study protocol	NA
Data collection	Retrospective study. Samples collected at the Pathology Department of the University Hospital La Paz (Madrid, Spain)
Outcomes	Tumor recurrence.
